# Morphogenesis of a chiral liquid crystalline droplet with topological reconnection and Lehmann rotation

**DOI:** 10.1038/s41598-024-58054-2

**Published:** 2024-03-31

**Authors:** Jun Yoshioka, Yuki Ito, Koji Fukao

**Affiliations:** Department of Physical Sciences, Ritsumeikan University, 1-1-1 Noji-Higashi, Kusatsu, Shiga 525-8577 Japan

**Keywords:** Liquid crystals, Fluid dynamics

## Abstract

Morphogenesis is a hierarchical phenomenon that produces various macroscopic structures in living organisms, with high reproducibility. This study demonstrates that such structural formation can also be observed in a chiral liquid crystalline droplet under a temperature gradient. Through specific control of the temperature change process, we were able to switch the final structure obtained as a result of the formation via the appearance and reconnection of loop defects in the transient state during structure formation. Simultaneously, the existence of the gradient resulted in a characteristic rotational phenomenon called Lehmann rotation, which was prominently induced in the transient state. By demonstrating three-dimensional measurements of the flow field, we revealed the existence of Marangoni convection in the state. Consequently, it is indicated that the convection results in high-speed Lehmann rotation and large structural deformation with topological changes, thereby playing a significant role in the structure formation.

## Introduction

Morphologies of living organisms are formed via ordering and disordering processes with various size scales, as observed in the segmentation and differentiation of eggs, or the metamorphosis of insects^1,2^. Morphogenesis is a highly controlled phenomenon that generates various macroscopic structures with a high reproducibility. A key factor in this phenomenon is the appearance of a concentration gradient, indicating that a potential gradient is required for morphogenesis. The present study demonstrates that such a structural formation process under a gradient can also be observed in liquid crystalline (LC) systems, which exist as components of living organisms.

An example of the macroscopic structure formed by LC has been observed in droplets composed of nematic (N), smectic, or cholesteric (Ch) phase^3–20^. Notably, the chiral LC system of the Ch droplets exhibits a wide range of structural diversity. Various structures with three-dimensional helices and point, line, and loop defects are formed, depending on the droplet size, helical pitch length, elastic constants, and anchoring constants^3,11–20^. However, even when these parameters, which are considered to determine an equilibrium state, are successfully controlled, the realised structure may not be uniquely determined in real systems. Reportedly, the internal director field in the droplet can show different states, although the experimental conditions are controlled to be identical^13,15,18,20^. One may consider that this structural discrepancy is attributed to variations in the structure formation process. Consequently, it is hypothesised that control of the process should result in complete structural control of the Ch droplet. In this study, we fabricated disk-shaped droplets sandwiched by substrates via a cooling process from an isotropic liquid (I) phase under a constant temperature gradient. The existence of this gradient enables gradual growth of the domain with LC order from the low-temperature side of the droplet^21^. This controlled growth should enable control of the final structure of the Ch droplets obtained by the cooling process.

The existence of a gradient, such as a thermal, electric, or chemical potential gradient in the Ch LC, may yield a rotation of the director field; this characteristic phenomenon is called Lehmann rotation^22–25^. The phenomenon was first discovered by Lehmann in the Ch LC under a temperature gradient in 1900 but was not reproduced for more than a century after the discovery. In this situation, as a reproduction of the Lehmann rotation, a steady rotation was observed in a Ch droplet under a temperature gradient by Oswald et al. in 2008^26^. Subsequently, this phenomenon was reproduced by several research groups^18,27–35^. As the mechanism of the rotational phenomenon, two possibilities have been proposed. The first is based on Leslie’s theory, which predicts the existence of direct coupling between director rotation and the temperature gradient in Ch LC^23,36^. The other mechanism is attributed to Marangoni convection resulting from the surface tension gradient; the rotation is driven by the material flow in the twisted director field^31,33^. To date, the rotational phenomenon has mainly been observed in droplets dispersed in specific liquid solvents^18,26–35^. In contrast, we used a system without solvents; a droplet directly exposed to air was used in this study. However, despite this difference, Lehmann rotation should also be observed in our system if the rotation is driven by any of the aforementioned mechanisms.

In this study, we demonstrate the structural formation processes of N and Ch droplets with an air interface in the presence of a temperature gradient. These processes are accompanied by characteristic dynamics, such as structural deformation, Lehmann rotation, and Marangoni convection. A comparison of the observation results for the Ch and N droplets clarifies the contribution of the existence or nonexistence of chirality to the dynamics. Based on experimental analyses of the director and flow fields, we report a possible description of the structure formation in these droplets.

## Results

### Structure formation of nematic and cholesteric droplets

**Figure 1:**
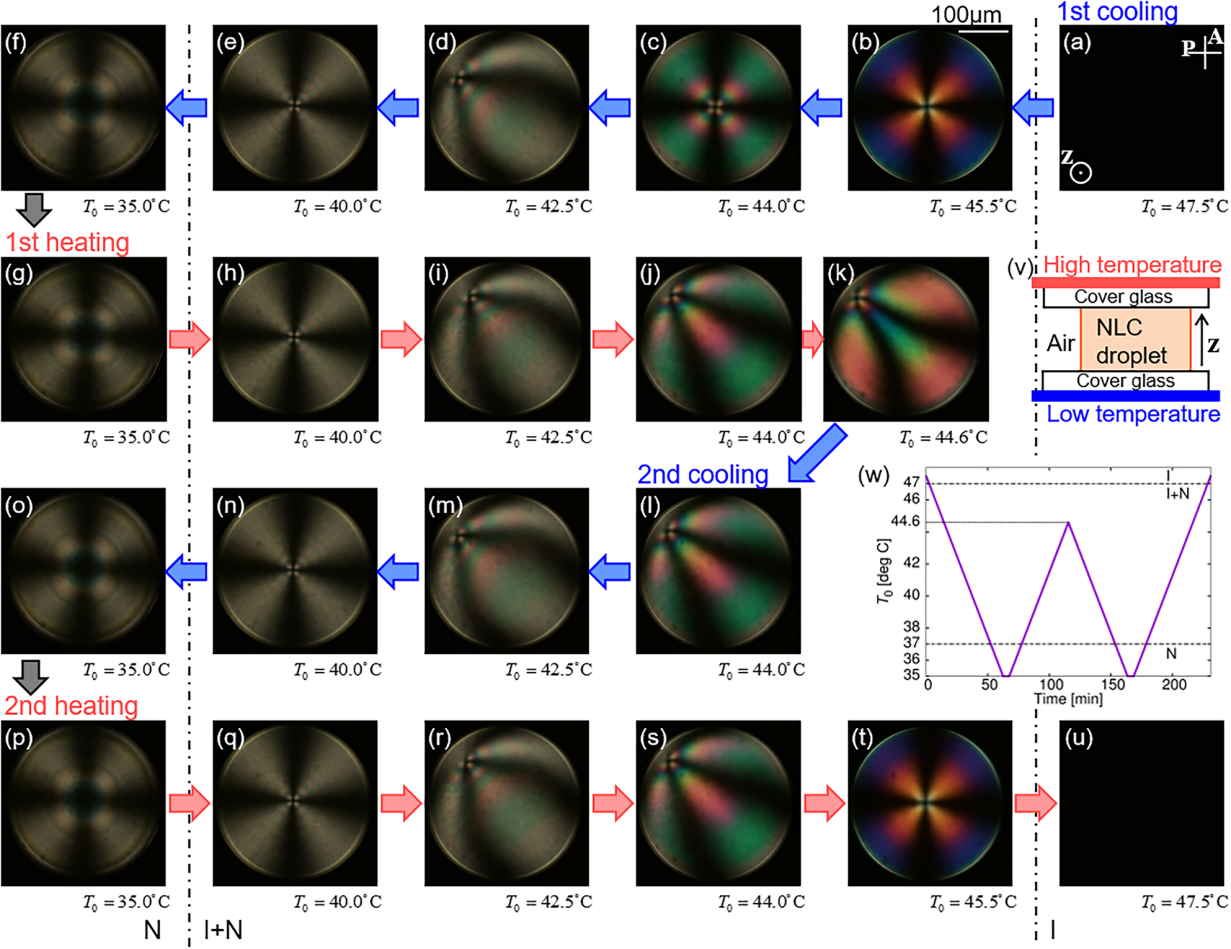
Structural formation of an N droplet. Temperature difference between upper and lower glass substrates was Δ*T* = 10K, and heating/cooling rate was 0.2 K/min. (**a**–**u**) show POM images at each *T*_0_, defined as the temperature at the droplet centre. A and P in (**a**) indicate analyser and polariser respectively, and the white bar in (**b**) 100 μm. Schematic image of experimental system is shown in (v), and the heating and cooling chart is in (w). The chart consists of two cooling and heating processes, which are called 1st and 2st coolings, and 1st and 2st heatings, respectively. The corresponding movies are available in Supplementary Videos 1–3.

First, by creating an N droplet, we applied a temperature gradient (see Fig. 1(v), the Methods section and Supplementary Note 2). In this study, *T*_0_ is defined as the temperature at the droplet centre, and Δ*T* is the temperature difference between the upper and lower glass substrates (For more detail about the determination of *T*_0_ and Δ*T*, see Supplementary Note 3 and ref.^37^). Keeping Δ*T* constant, we changed *T*_0_ according to the chart in Fig. 1(w), which consists of two cooling and heating processes. Starting from the I phase, we decreased *T*_0_ and observed the textural changes in the droplet using polarised microscopy (POM). In the 1st cooling process, a cross-texture was observed in the coexisting state of the I and N phases (I + N phase), as shown in Fig. 1b–e. This indicates that the director field has a radial alignment and a point defect exists in the droplet^18,23,38^. During the cooling process, a point defect first appeared at the droplet centre (Fig. 1b) and then moved away from the centre at a lower temperature (Fig. 1d). Subsequently, the defect returned to the droplet centre (Fig. 1e), and this configuration was preserved until the entire droplet transited into the N phase. A centred cross texture was also observed after the transition (Fig. 1f); the radial director alignment was also formed in the N phase^39^, as well as in the I + N phase.

After the 1st cooling process, the temperature was increased. In this 1st heating process, the point defect was located at the droplet centre at a lower *T*_0_ of less than ~ 40°C and moved away from the centre at a higher *T*_0_ as shown in Fig. 1g–k, as well as in the 1st cooling process. The 1st heating process ended with the off-centre state of the defect (Fig. 1k), after which the temperature decreased. In this 2nd cooling process, the point defect returned to the droplet centre in the I + N phase, as shown in Fig. 1l–n. Finally, a cross texture similar to that observed in the 1st cooling process was formed (Fig. 1o). Subsequently, the temperature was increased. In this 2nd heating process, the movement of the point defect similar to that in the 1st heating was observed, as shown in Fig. 1g–j and p–s. Finally, the point defect returned to the droplet centre, and the droplet transitioned into the I phase (Fig. 1t and u).

By defining *R* as the droplet radius and *R*_*c*_ as the distance of the point defect from the droplet centre, we measured *T*_0_ dependence of ${{R_{c} } \mathord{\left/ {\vphantom {{R_{c} } R}} \right. \kern-0pt} R}$ as shown in Fig. 3a. The temperature region wherein ${{R_{c} } \mathord{\left/ {\vphantom {{R_{c} } R}} \right. \kern-0pt} R}$ shows a non-zero value depends on whether the temperature protocol is cooling or heating, suggesting the existence of thermal hysteresis. ${{R_{c} } \mathord{\left/ {\vphantom {{R_{c} } R}} \right. \kern-0pt} R}$ in the 2nd cooling process agrees with ${{R_{c} } \mathord{\left/ {\vphantom {{R_{c} } R}} \right. \kern-0pt} R}$ in the 1st heating when *T*_0_ is relatively high ($T_{0} > \sim 43\;^\circ {\text{C}}$), and agrees with ${{R_{c} } \mathord{\left/ {\vphantom {{R_{c} } R}} \right. \kern-0pt} R}$ in the 1st cooling when *T*_0_ is relatively low ($T_{0} < \sim 43\;^\circ {\text{C}}$). Owing to the agreement in the cooling processes, the final structures obtained in the N phase in the 1st and the 2nd coolings were identical, as shown in Fig. 1f and o.

**Figure 2:**
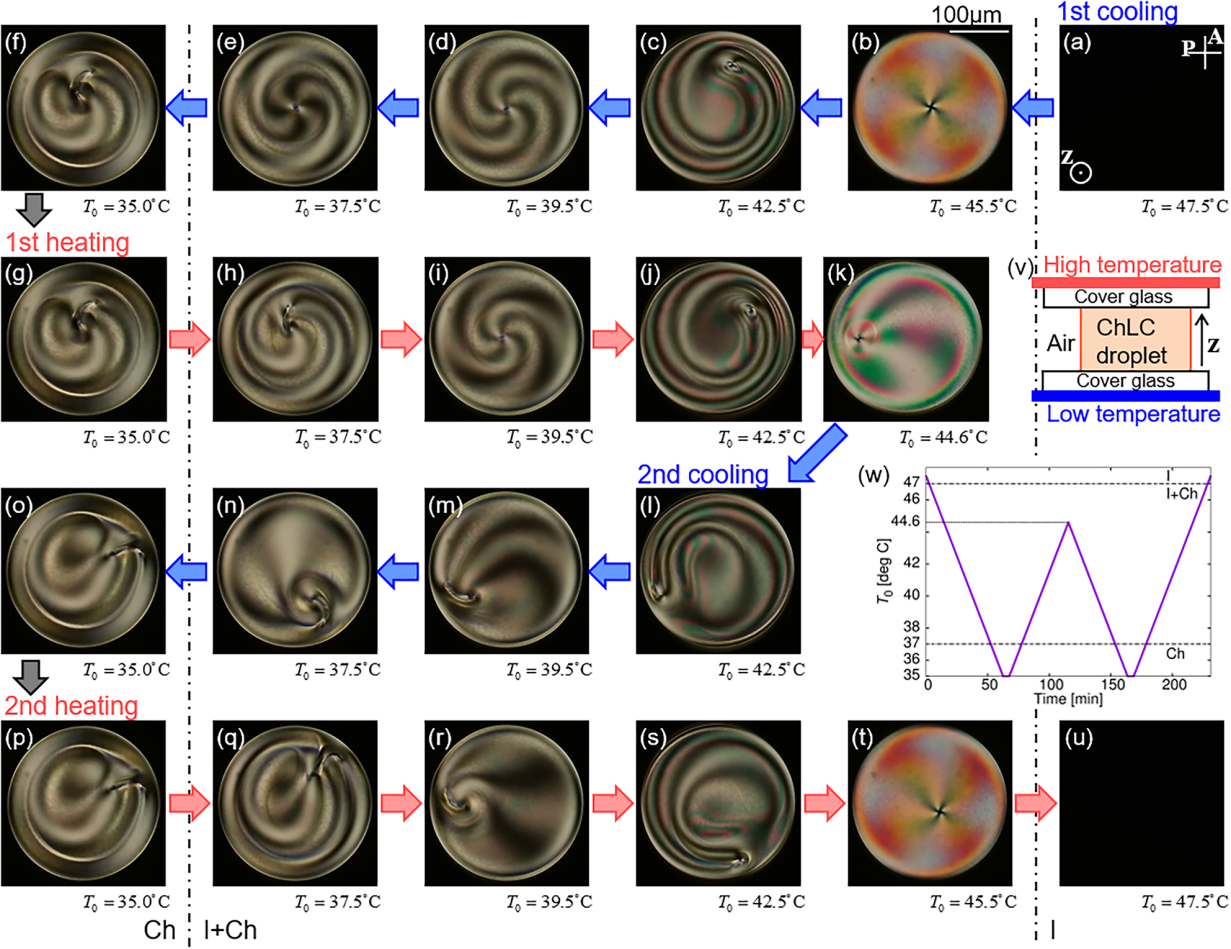
Structural formation of a Ch droplet. Temperature difference between upper and lower glass substrates was Δ*T* = 10K, and heating/cooling rate was 0.2 K/min. (**a**–**u**) show POM images at each *T*_0_, defined as the temperature at the droplet centre. A and P in (**a**) indicate analyser and polariser respectively, and the white bar in (**b**) 100μm. Schematic image of experimental system is shown in (v), and the heating and cooling chart is in (w). The chart consists two cooling and heating processes, which are called 1st and 2st coolings, and 1st and 2st heatings, respectively. The corresponding movies are available in Supplementary Videos 4–6.

Subsequently, we observed the Ch droplets in a manner similar to that described previously. In the 1st cooling process, the centred cross texture was first formed in the coexistence phase (I + Ch phase), as shown in Fig. 2b. The appearance of the texture indicates the formation of a point defect as well as an N droplet. The defect moved away from the centre during the cooling process, as shown in Fig. 2c. In contrast to N droplet, the point defect exhibited a clockwise circular motion (see Supplementary Video 4). As the temperature decreased further, the defect returned to the droplet centre, and the circular motion stopped (Fig. 2d). This configuration was preserved just before the entire droplet transitioned into the Ch phase. As the transition occurred, a comma-like texture formed near the droplet centre, as shown in Fig. 2f. In this study, we refer to a Ch droplet with this texture as the ‘Type-A’ state.

In the 1st heating process, the comma-like texture was preserved just after the transition into the I + Ch phase, as shown in Fig. 2h, whereas at higher *T*_0_ the texture deformed into a cross-like texture, again indicating the formation of a point defect at the droplet centre (Fig. 2i). Subsequently, the defect moved away from the centre and exhibited a clockwise circular motion, as shown in Fig. 2j and Supplementary Video 5. The 1st heating process ended with an off-centre circulating state of the point defect (Fig. 2k). In the following 2nd cooling process, the cross texture gradually deformed into a comma-like texture, as shown in Fig. 2k–n, and it did not return to the droplet centre. The circular motion continued after the texture deformed into a comma-like shape, whereas its direction changed from clockwise to counterclockwise (see Supplementary Video 5). As the transition into the Ch phase occurred, the comma-like texture remained near the droplet edge, as shown in Fig. 2o; we refer to this state as ‘Type-B’, distinguished from Type-A (Fig. 2f). In contrast to N droplet, the final structure of Ch droplet depends on whether it is obtained during the 1st or 2nd cooling process. Since both type-A and -B states hold under the absence of the temperature gradient, they are considered as the stable or one of the metastable structures at equilibrium condition (see Supplementary Note 4). In the 2nd heating process, the comma-like texture gradually deformed into a cross texture, and the direction of the circular motion changed from counterclockwise to clockwise (Fig. 2p–s and Supplementary Video 6). Subsequently, the cross-texture defect moved to the droplet centre before the transition into the I phase (Fig. 2t and u).

**Figure 3:**
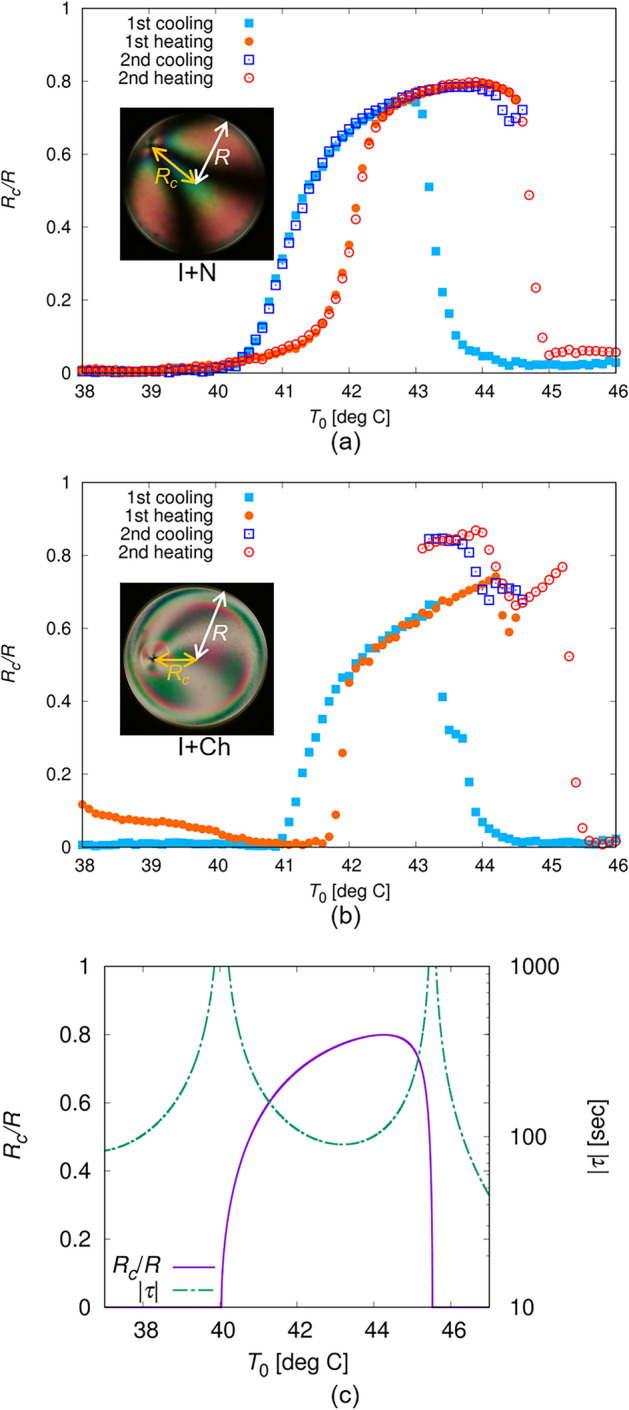
Temperature dependences of ${{R_{c} } \mathord{\left/ {\vphantom {{R_{c} } R}} \right. \kern-0pt} R}$ in N (**a**) and Ch (**b**) droplets. *R* is defined as the droplet radius, and $R_{c}$ as the distance between the point defect and the droplet centre; the definitions of them are illustrated in the insets of (**a**) and (**b**). The measurements of ${{R_{c} } \mathord{\left/ {\vphantom {{R_{c} } R}} \right. \kern-0pt} R}$ were performed under the temperature (*T*_0_) chart shown in Figs. 1(w) and 2(w), consisting of 1st cooling and heating, and 2nd cooling and heating processes, where Δ*T* was 10K. In (**b**), the data is not shown when *T*_0_ is lower than 43 °C in the 2nd cooling and heating processes. In this period, the point defect transformed into the structure with the comma-like texture (Fig. 2(l)–(n) and (q)–(s)), where *R*_*c*_ cannot be well-defined. (**c**) is the *T*_0_ dependence of steady-state solution of ${{R_{c} } \mathord{\left/ {\vphantom {{R_{c} } R}} \right. \kern-0pt} R}$ and the absolute value of *τ*, obtained by theoretical analysis. *h* and *R* were assumed to be 100 μm and 150 μm, respectively, and *V*_*s*_ was 1.0 μm/s. The rotational viscosities were assumed as *γ*_1_ = *γ*_2_ = 0.030 Pa·s^40,41^, and the elastic constants were *K*_1_ = *K*_3_ = 4.0 pN and *K*_2_ = 2.0 pN^42^.

*T*_0_ dependence of ${{R_{c} } \mathord{\left/ {\vphantom {{R_{c} } R}} \right. \kern-0pt} R}$ on the Ch droplets is shown in Fig. 3b. It demonstrates thermal hysteresis in the 1st cooling and heating processes, as well as in the N droplet. However, the ${{R_{c} } \mathord{\left/ {\vphantom {{R_{c} } R}} \right. \kern-0pt} R}$ in the 2nd cooling did not agree ${{R_{c} } \mathord{\left/ {\vphantom {{R_{c} } R}} \right. \kern-0pt} R}$ in the 1st heating or cooling process, and the point defect gradually changed into a structure with a comma-like texture, as described previously (Fig. 2k–n). After the structural change we could no longer define *R*_*c*_, so that the data is absent in the region with comma-like texture in Fig. 3b. These hysteresis behaviours were not observed in the N droplet, and the observation results in Figs. 2 and 3b show that the structure formation in the Ch droplet followed different kinetic pathways in the 1st and the 2nd cooling processes, respectively. This difference in the pathway results in structural differences observed in the Ch phase (Type-A and Type-B states).

**Figure 4:**
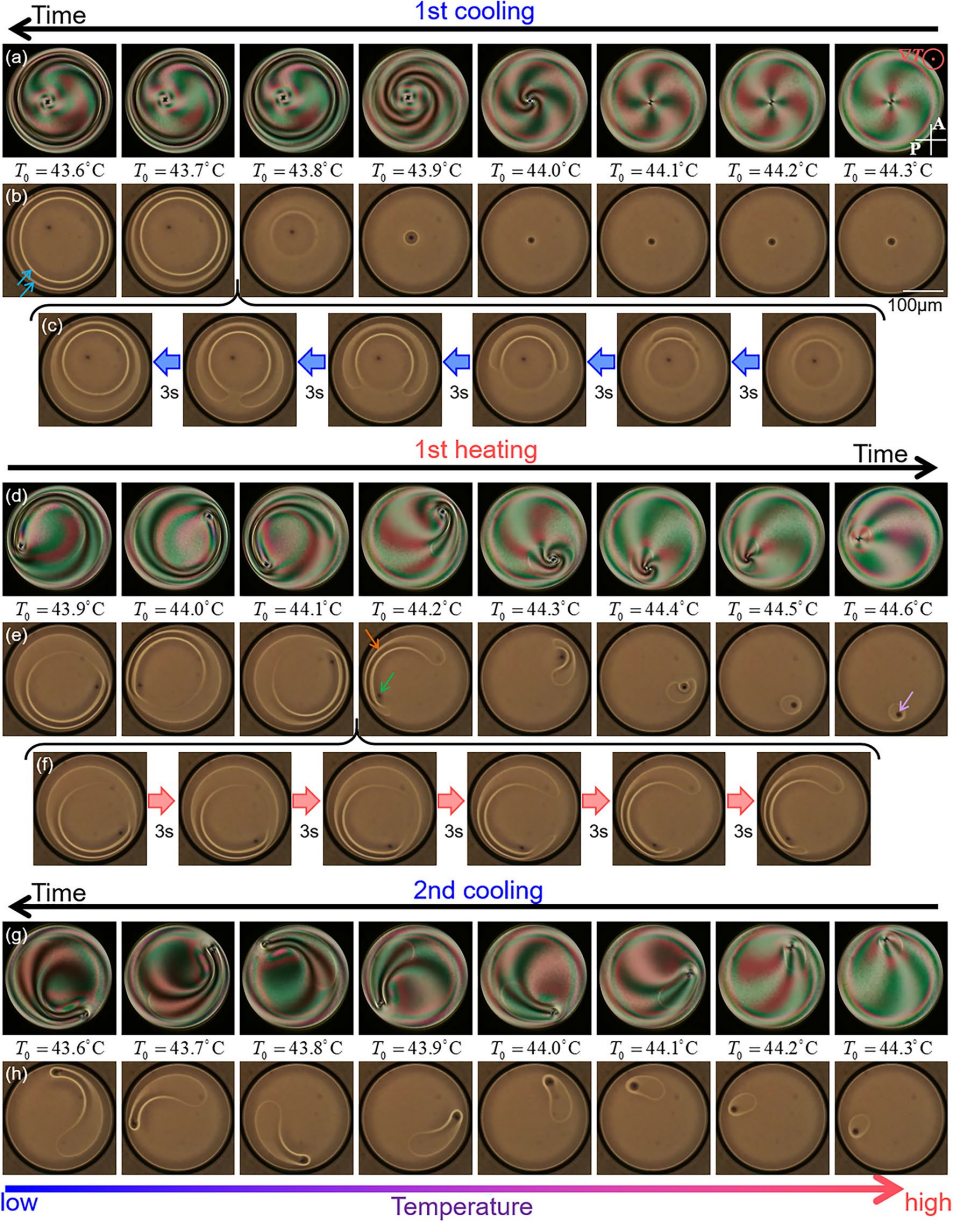
Observation of the formation, deformation and reconnection of defect structures. (**a**), (**d**) and (**g**) are POM images, and the others are the observation results without polarisers. Red symbol in (**a**) indicates the direction of the temperature gradient. A and P in (**a**) indicate analyser and polariser respectively, and the white bar in (**b**) 100 μm. In (**a**, **b**, **d**, **e**, **g** and **h**), temperature difference in each figure is 0.1K and the cooling/heating rate 0.2K/min, so that the time difference in each figure is 30 s. (**c** and **f**) are displayed with higher time resolution (3 s each), showing the detailed change of the texture when the loop defect is formed and reconnected. During the 1st cooling process, the double loop defects, indicated by the aqua arrows in (**b**), are formed. In the end of the 1st heating process, the double loop defects are transformed into a single loop, as shown by the orange arrow in (**e**). In this instance the point defect (green arrow) is outside of the loop. After that, the point defect goes inside the single loop as indicated by the purple arrow. The corresponding movies are available in Supplementary Videos 4–8.

The cross texture observed in the Ch droplets was twisted. In the 1st cooling process, this twist drastically progressed in the temperature range between *T*_0_ = 44.1 and 43.8 °C as shown in Fig. 4a. In addition, by observing the droplet without polarisers, we discovered that double-ring textures were formed just after the drastic progress of the twist, as shown in Fig. 4a–c and Supplementary Videos 4 and 7. The appearance of rings suggests the formation of double-loop defects, as reported in the literature^13,16^.

Reconnection of the defect structure was observed at the end of the 1st heating process. As shown in Fig. 4e and f, the double loops changed into a single loop in the temperature between *T*_0_ = 44.1 and 44.2 °C (see also Supplementary Videos 5 and 8). Immediately after reconnection, the point defect observed as a dark point was outside the single-loop defect, whereas the defect then entered the inside of the loop, as shown in Fig. 4e. Thus, a topological change in the defect structure was induced, resulting in a structural difference in the Ch phase (type-A and B states). The single loop was gradually elongated with a point defect inside the loop in the 2nd cooling process (Fig. 4h), and it never followed the structural change in the 1st heating or cooling processes.

### Director rotation in cholesteric droplet under temperature gradient

**Figure 5:**
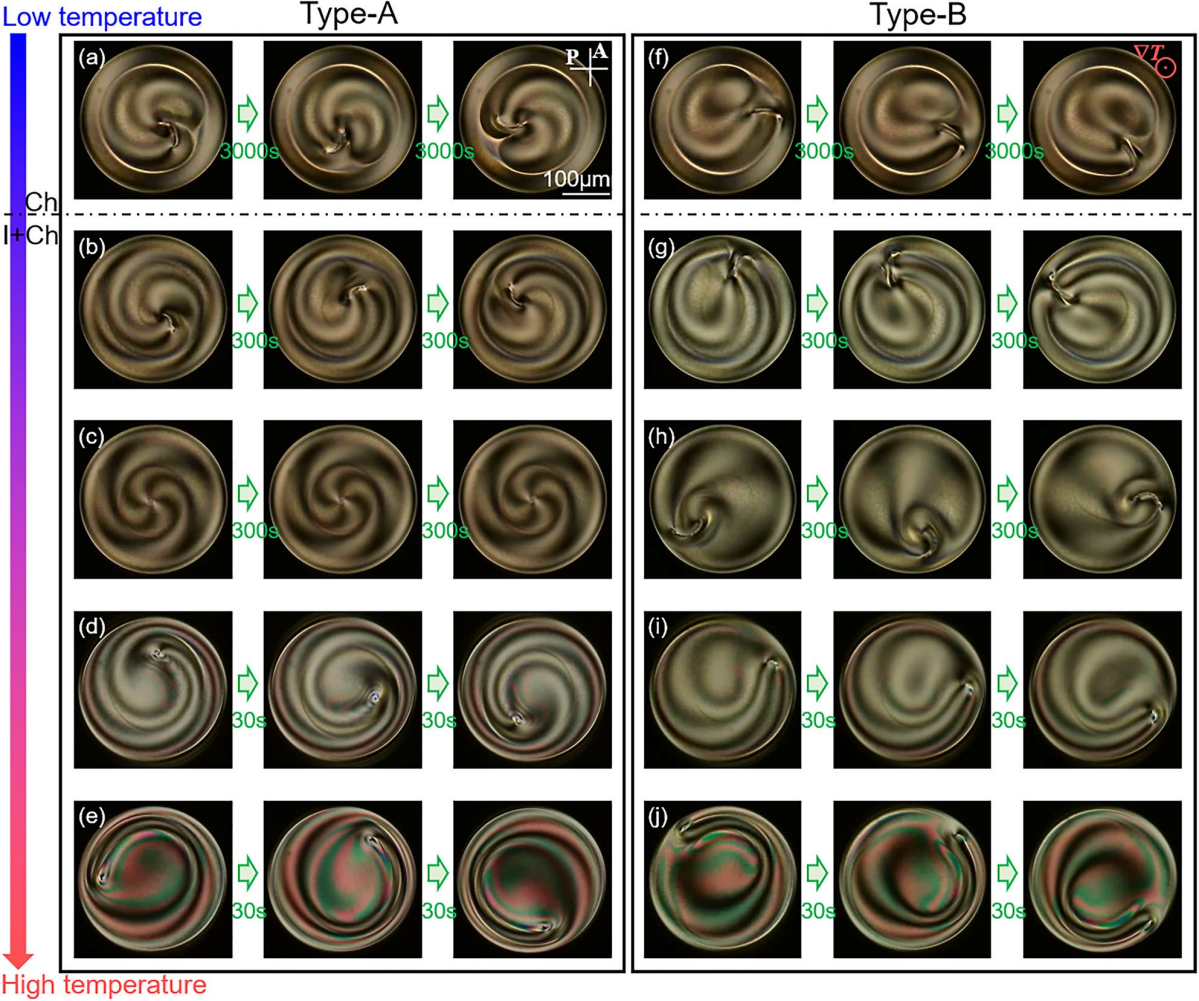
Director rotations under steady states. The time evolutions of POM images are shown. A and P in (**a**) indicate analyser and polariser respectively, and the white bar 100 μm. Red symbol in (**f**) indicates the direction of the temperature gradient. Δ*T* is set to be 10 K, and *T*_0_ is 35.0 °C in (**a**) and (f), 37.5 °C in (**b**) and (**g**), 38.5 °C in (**c**) and (**h**), 42.0 °C in (**d**) and (**i**) and 43.0 °C in (**e**) and (**j**), respectively. (**a**) and (**f**) are observed in the Ch phase, and the others are in the I + Ch phase. Time interval in each figure is 3000 s in (**a**) and (**f**), 300 s in (**b**), (**c**), (**g**) and (**h**) and 30 s in (**d**), (**e**), (**i**) and (**j**). Clockwise rotation was observed in (**a**), (**d**)–(**f**), (**i**) and (**j**), while counter-clockwise was in (**b**), (**g**) and (**h**). The corresponding movies are available in Supplementary Video 9.

Director rotation was driven in the Ch droplet not only under an unsteady state with a change in *T*_0_ but also under a steady state. Keeping both *T*_0_ and Δ*T* constant, we observed that the steady clockwise rotation of the director field was driven in both the Type-A and Type-B Ch states, as shown in Fig. 5a and f (see also Supplementary Video 9(a) and (f)). Since the structural change was not observed during the rotation, we should consider that these rotating droplets were in the steady state. The rotational speed was proportional to Δ*T* (see Supplementary Note 5), as is often reported for Ch droplets with Lehmann rotation^18,26–28,35^. This indicates that the rotations observed in the Ch phase were driven by the application of a temperature gradient.

**Figure 6:**
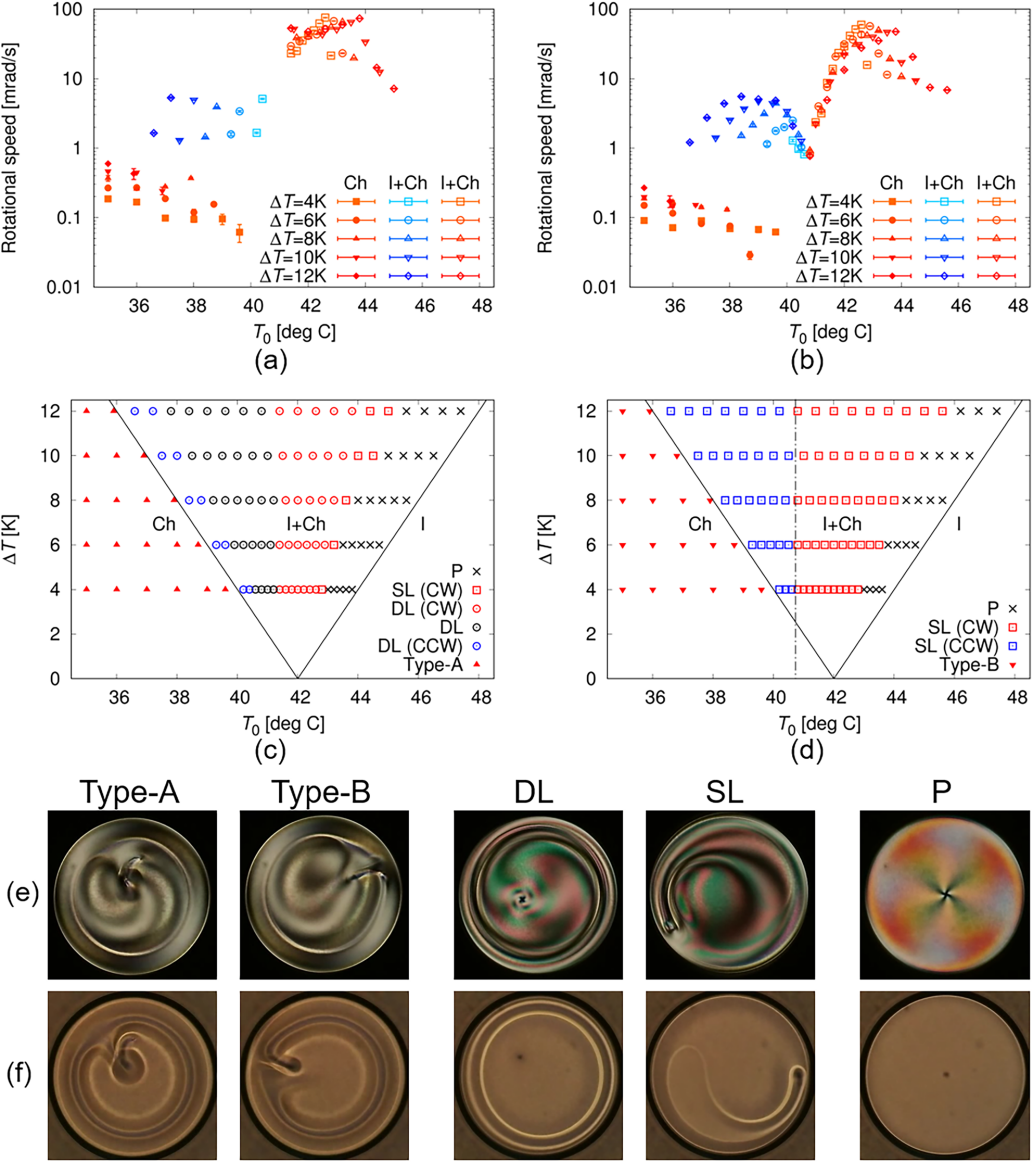
Temperature dependence of rotational speed and state diagram. (**a**) and (**b**) show the dependences of the rotational speed on *T*_0_ and Δ*T* in the case of Type-A and B, respectively. The symbols with warm colours indicate the appearance of clockwise (CW) rotation, and cold colours indicate counter-clockwise (CCW). The data obtained in the Ch and I + Ch phases are shown by closed and open symbols, respectively. (**c**) and (**d**) are the state diagrams under constant *T*_0_ and Δ*T* in the case of Type-A and B Ch droplets, respectively. Based on the observation images under crossed polarizers and without polarisers, the structures were categorised into five groups as shown in (**e**) and (**f**). The structure in the Ch phase is categorised into Type-A and B, which are obtained at the end of the 1st and 2nd cooling processes, respectively. The I + Ch phase is categorised into three groups: DL, SL and P states. Only a point defect exists in the P state, while in the DL and SL states double and single loop defects are formed, respectively. The point defect can exist also in the DL and SL states, while it transforms into the structure with comma-shaped texture at lower temperature (Fig. 5 (**b**) and (**g**)–(**i**)). The observation was first performed under a constant Δ*T* in the Type-A or Type-B state, and the following observations were performed in stepwise increase of *T*_0_. Changing Δ*T* and repeating these observations, we obtained the results of (**a**), (**b**), (**c**) and (**d**). Here, the red and blue symbols indicate that CW and CCW rotations were observed respectively, and the black symbols indicate that no rotation was observed. In the I + Ch phase in (**d**), the direction of the rotation reverses at the broken line: this indicates that the direction is controlled by *T*_0_.

Subsequently, keeping Δ*T* constant and increasing *T*_0_, we obtained the I + Ch phases for Type-A and Type-B, respectively. In these situations, we observed the droplets under constant *T*_0_ and Δ*T*. Just above the transition temperature from the Ch to the I + Ch phase, rotation was observed in both the Type-A and B as shown in Fig. 5b and g. Herein, both showed counter-clockwise rotation, reversed from the rotation observed in the Ch phase (see Supplementary Video 9(a), (b), (f), and (g)). In addition, the rotational speed in the I + Ch phase is one order of magnitude higher than that in the Ch phase (closed symbols with warm colours and open symbols with cold colours in Fig. 6a and b). As *T*_0_ increased further, in the case of Type-A, a four-fold symmetric texture with the centred point defect was formed and the rotation stopped, whereas in Type-B, the structure with the off-centred defect was preserved and the director field continued rotating, as shown in Fig. 5c and h (see also Supplementary Video 9(c) and (h)). With increasing *T*_0_, the rotational direction again reversed: in both cases of Type-A and B, the clockwise rotation was observed as shown in Fig. 5d, e, i and j and Supplementary Video 9(d), (e), (i) and (j); simultaneously, the rotational speed increased by one order of magnitude (open symbols with cold and warm colours in Fig. 6a and b). Heating the droplet further from this state, we observed that the four-fold symmetric texture shown in Fig. 2b was formed in both Types-A and B; in this state, rotation was never observed.

By changing Δ*T* and repeating the above observation, we created state diagrams for the rotation, as shown in Fig. 6c and d. The red and blue symbols indicate that clockwise and counter-clockwise rotation was observed in the diagrams, respectively, and the black symbols indicate that no rotation was under the corresponding conditions. In addition, based on the structural difference of the droplet, the states in the I + Ch phase were categorised into the three groups: DL state with the double-loop defects, SL state with a single-loop defect, and the P state with only a point defect. In the regions of no rotation (black symbols), four-fold symmetric textures, as shown in Fig. 2b and d, were always observed. The appearance of this texture under the POM observation indicates the existence of the director field with circular symmetry^18,23,38,39^. In this situation, director rotation cannot be driven unless the director field is deformed. Thus, director rotation was always observed in this experiment unless a structure with circular symmetry was formed.

Notably, the rotational direction is determined by *T*_0_ in the I + Ch phase, as seen in the type-B state diagram of Fig. 6d. Herein, we considered that this is attributed to the peculiar property of the surface tension near the phase transition temperature, and to the fact that the direction of the Marangoni flow depends on the surface tension gradient^43–45^. In the N LC material of 7CB used in this study (see Methods section), it was found that the flow direction depends on the temperature owing to the drastic change in surface tension near the I–N transition point^21,46^. By measuring the surface tension of the Ch LC sample used in this study, we confirmed that its temperature dependence is similar to that of 7CB (see Supplementary Note 6). As the microscopic origin for the singular properties about the surface tension, the contribution of the orientational order parameter in the surface has been discussed in refs.^47–50^, while the origin remains to be solved. Anyway, we consider that the inversion of the director rotation in the I + Ch phase might be due to the inversion of the Marangoni convection. To validate this notion, we measured the flow field inside the droplet, as described in the next section.

### Flow field in the droplet under temperature gradient

**Figure 7:**
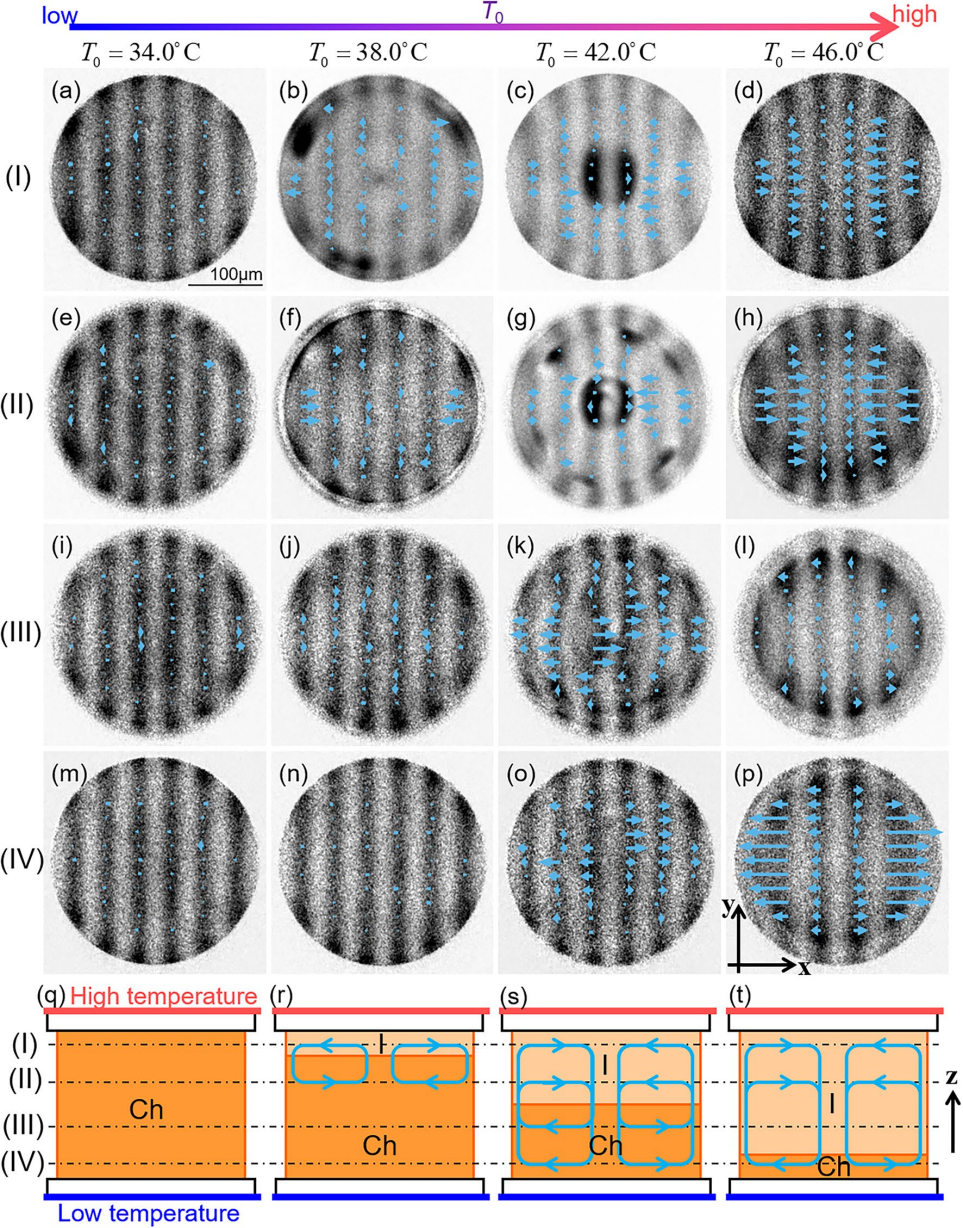
Measurement results of the flow field. The measurement was performed in the focal planes (I)–(IV) shown in (**q**). The planes (I) and (II) are 15 and 35μm far from the high-temperature side substrate respectively, and (III) and (IV) are 35 and 15 μm from the low-temperature side. For (I) and (II), the light was incident from the high-temperature side, and for (III) and (IV), the light was from the low-temperature side. The measurement was performed under four different temperatures *T*_0_ of 34.0, 38.0, 42.0 and 46.0 °C, and the temperature difference Δ*T* was set to be 12K. The droplet was in the Type-A Ch state when *T*_0_ = 34.0 °C, and I + Ch phase for otherwise, as illustrated in (**q**–**t**). In (**a**–**p**), the distribution of the flow velocity in each focal plane and *T*_0_ is drawn together with the photo-bleached pattern, which is normalised by the fluorescence image before the bleaching. The black bar in (**a**) indicates 100 μm.

**Figure 8:**
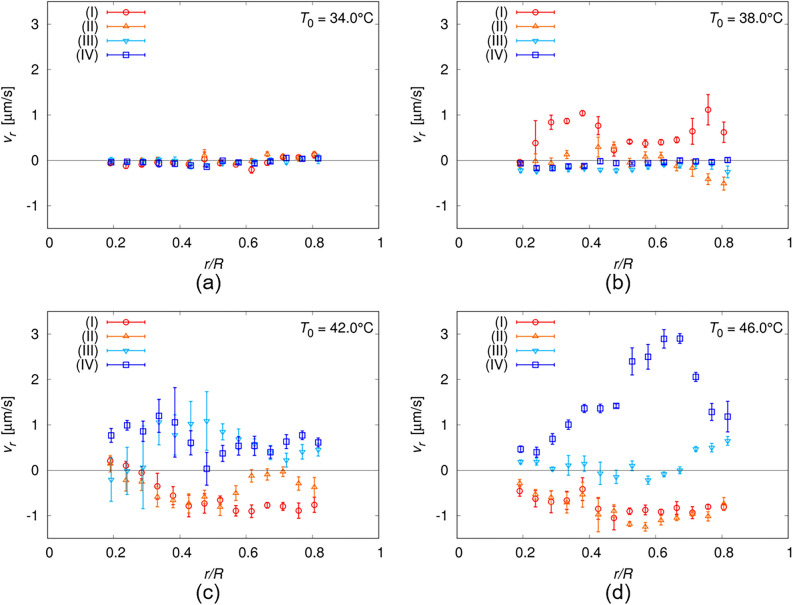
Temperature dependence of radial flow velocity *v*_*r*_. The velocity was estimated by the measurement results shown in Fig. 7. The measurement was performed in the four focal planes of (I)–(IV) illustrated in Fig. 7q under four different temperatures *T*_0_ of 34.0, 38.0, 42.0 and 46.0 °C, as shown in (**a**–**d**). *v*_*r*_ is plotted with respect to *r*/*R*, where *r* is radial coordinate, and *R* is the droplet radius. The positive value of *v*_*r*_ indicates the outward flow, and negative *v*_*r*_ indicates inward.

The flow field was measured using the fluorescence photobleaching method, as shown in Fig. 7. The use of a confocal microscope enabled us to three-dimensionally analyse the field. Assuming that the flow was circularly symmetric, we estimated the radial flow velocity *v*_*r*_ for the measurement results obtained in each focal plane, as shown in Fig. 8 (see the Methods section and Supplementary Note 7). When *T*_0_ was relatively high in the I + Ch phase, an inward flow was observed near the high-temperature side of the substrate, whereas an outwards flow was observed near the low-temperature side, as shown in Figs. 7c, d, o, p, 8c, and d. These observations indicated the existence of convection inside the droplet, as shown in Fig. 7s and t. In contrast, when *T*_0_ was relatively low in the I + Ch phase, the measurement result near the high-temperature side of the substrate showed the existence of an outwards flow (Figs. 7b and 8b), in contrast to the inward flow observed at a higher *T*_0_ (Figs. 7c, d, 8c, and d). This inversion indicates that the direction of the convection was inverted depending on *T*_0_, and this dependence is consistent with the expectation of the direction of the Marangoni convection associated with director rotation in the previous section. Therefore, the measurement results obtained in this study support the notion that the director rotation in the I + Ch phase is driven by convection. Owing to the coupling between the director and the flow fields, the director rotation is induced as described in refs^31,33^.

In the I + Ch phase with a low *T*_0_, flow was hardly observed near the low-temperature side of the substrate (Figs. 7n and 8b). This indicates that convection was localised near the high-temperature side of the substrate, as shown in Fig. 7r, in contrast to the convection induced at a higher *T*_0_ (Fig. 7s and t). This difference in the location of convection results in a large difference in the rotational speed of the director field of the I + Ch phase. As mentioned in the previous section, the speed decreased by one order of magnitude when *T*_0_ decreased in the phase (Fig. 6a and b). This drastic decrease can be attributed to the localisation of the Marangoni convection near the high-temperature region in the droplet: owing to the localisation, the flow generates the rotational torque only in the narrow region near the I-Ch interface when *T*_0_ is relatively low (see Fig. 7r), and the rotation is strongly suppressed by the dissipation produced in the region without flows.

Under the assumption that the rotation is induced by the Marangoni flow, we can give a rough estimation for the rotational speed. Considering the advection effect of the director field according to refs.^31,33^, the angular velocity of the rotation can be estimated as $\omega \sim {{2\pi V} \mathord{\left/ {\vphantom {{2\pi V} {P_{0} }}} \right. \kern-0pt} {P_{0} }}$, where *V* is a characteristic flow speed and *P*_0_ is the helical pitch length. Assuming that *P*_0_ is ~ 50 µm (see Method section) and *V* is the order of 10^–1^–10^0^ µm/s in the I + Ch phase from Fig. 8, *ω* can be estimated to be ~ 10^1^–10^2^ mrad/s. This is overlapping with the measured rotational speed in the I + Ch phase as shown in Fig. 6a and b, whereas ranges slightly higher than the measurement results. Since the factors inhibiting the rotation, such as viscous dissipation and the deformation of the director field, were neglected in the estimation, it necessarily results in an overestimation. Thus, it is sufficiently reasonable to consider that the rotation is mainly induced by the flow, also from the quantitative discussion about the rotational speed.

In the Ch phase, the flow was barely observed anywhere in the droplet, as shown in Figs. 7 a, e, i, m, and 8a. This is consistent with the rotational speed: the speed in the Ch phase was one or two orders of magnitude lower than that in the I + Ch phase (Fig. 6a and b). It is difficult to determine the mechanism of rotation in the Ch phase based on the results obtained in this study. One potential explanation for the mechanism is the direct coupling effect predicted by Leslie^23,36^. However, Marangoni convection remains a plausible candidate as well; the weak flow, which was not detected by our measurements owing to insufficient resolution, might result in slow rotation. As seen here, in the rotating Ch LC droplet under the temperature gradient, it is generally difficult to discuss in detail about the contribution of the Leslie’s thermomechanical coupling effect, separately from the possibility that the rotation is induced by other factors, such as the flow. How the existence or non-existence of the coupling effect should be verified is still a problem to be solved.

As described in this section, the flow property drastically changed, depending on the temperature. Unfortunately, we cannot clarify the reason why the drastic change was induced in the present situation. For the clarification, detailed measurement and discussion about the distribution of the temperature and the surface tension in the droplet and the energy dissipation due to the anisotropic viscosity of LC would be necessary.

## Discussion

**Figure 9:**
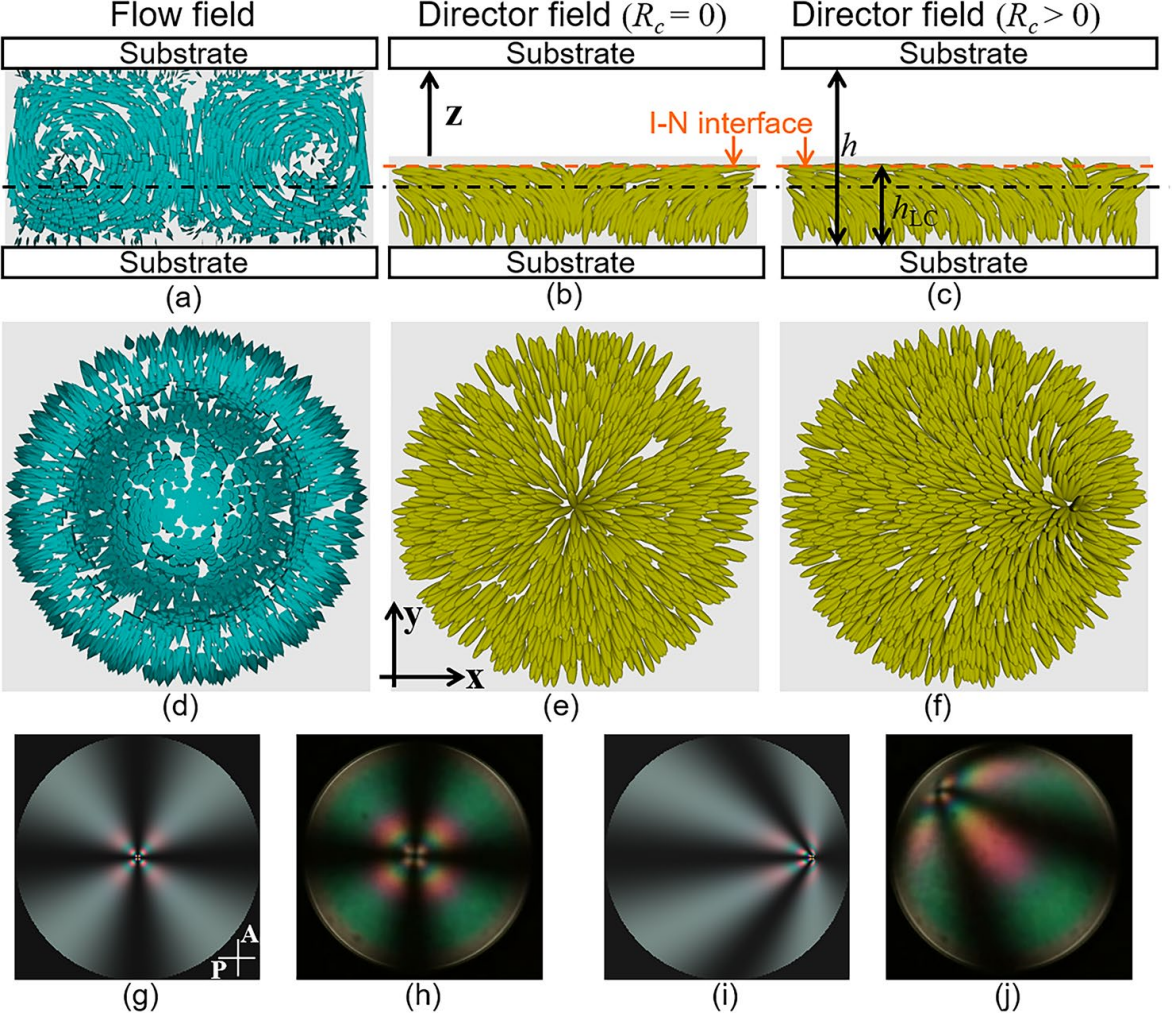
Analysis of flow and director fields in I + N phase. (**a**) and (**d**) represent the flow field depicted based on Eq. (1); (**b**), (**c**), (**e**) and (**f**) represent the director fields based on Eqs. (2) and (3). (**a**–**c**) show the fields at the plane including the central axis of the droplet, and (**d**–**f**) at the plane indicated by the broken line in (**a**–**c**). (**g**) and (**i**) are the light transmission intensity profiles calculated by the Jones matrix method under the assumption of Eqs. (2) and (3). P and A in (h) indicate polariser and analyser, respectively. ${{R_{c} } \mathord{\left/ {\vphantom {{R_{c} } R}} \right. \kern-0pt} R}$ is set to be 0 in (**b**), (**e**) and (**g**), and to be 0.7 in (**c**), (**f**) and (**i**). (**h**) and (**j**) are POM images obtained in the I + N phase.

Based on the experimental results, we discuss the deformation process of the director fields induced by flow. First, we consider the temperature dependence of *R*_*c*_, indicating the distance of the point defect from the droplet centre. Since ${{R_{c} } \mathord{\left/ {\vphantom {{R_{c} } R}} \right. \kern-0pt} R}$ shows a similar behaviour in both N and Ch droplets, except for the hysteresis property (Fig. 3a and b), we should consider that there is a common mechanism about the migration of the point defect apart from the droplet centre. For simplicity, here we consider the situation in N LC droplet. When *T*_0_ was relatively high in the coexistence phase, convection occurred in the entire droplet, as shown in Fig. 7s and t. In the similar way with refs.^31,38,39,44,45^, where the convection inside the droplet is discussed, we designed the trial functions for the flow velocity **v** = (*v*_*r*_, *v*_*ϕ*_, *v*_*z*_) as shown below:

1 $$\begin{gathered} v_{r} = 16V_{s} r\left( {1 - \frac{{r^{2} }}{{R^{2} }}} \right)\frac{z(h - z)(h - 2z)}{{h^{4} }} \hfill \\ v_{\phi } = 0 \hfill \\ v_{z} = - 16V_{s} \left( {1 - \frac{{2r^{2} }}{{R^{2} }}} \right)\frac{{z^{2} (h - z)^{2} }}{{h^{4} }}, \hfill \\ \end{gathered}$$

where we used cylindrical coordinates whose origin is set at the centre of the bottom plane of the droplet, as the present droplet has rotational symmetry. *R* and *h* are the droplet radius and height, respectively. *V*_*s*_ is the characteristic velocity; in the central plane of the $z = {h \mathord{\left/ {\vphantom {h 2}} \right. \kern-0pt} 2}$, *v*_*z*_ is − *V*_*s*_ and *V*_*s*_ at the centre (*r* = 0) and circumference (*r* = *R*), respectively. Equation (1) satisfies the incompressible (∇·**v** = 0) and non-slip conditions at the substrates (*v*_*r*_ = 0 for *z* = 0, *h*)^43^. The influx and outflux are zero at the droplet surface (*v*_*r*_ = 0 for *r* = *R*, *v*_*z*_ = 0 for *z* = 0, *h*). *v*_*ϕ*_ is assumed to be zero, because no rotational flow is considered to be induced in the N LC droplet. The velocity field described by Eq. (1) is depicted in Fig. 9a and d.

The director should align along the z-axis at the substrate owing to the strong homeotropic anchoring condition (see Methods). The side surface of the air interface also exhibits homeotropic anchoring^5^, whereas the interface between the I and LC phases is planar^51,52^. Considering these boundary conditions, radial alignment of the director should be realised in the I + N phase, as shown in Fig. 9b and e. The trial functions for similar situations have already been reported in refs.^38,39^. Based on these reports, we assumed the director field in the N droplet to be

2 $$\begin{gathered} n^{\prime}_{r} = \frac{{R^{\prime} + R_{c} \cos \phi^{\prime}}}{R}\sin \theta_{n} \hfill \\ n^{\prime}_{\phi } = - \frac{{R_{c} \sin \phi^{\prime}}}{R}\sin \theta_{n} , \hfill \\ n_{z} = \cos \theta_{n} \hfill \\ \end{gathered}$$

where we used the cylindrical coordinates of (*r*′, *ϕ*′, *z*). The parameters *θ*_*n*_ and *R*′ are described as

3 $$\begin{gathered} \theta_{n} = \frac{\pi }{2}\frac{z}{{h_{{{\text{LC}}}} }}\sqrt {\frac{{r^{\prime}}}{{R^{\prime}}}} \sum\limits_{k = 0}^{2} {\left( {1 - \sqrt {\frac{{r^{\prime}}}{{R^{\prime}}}} } \right)}^{k} \hfill \\ R^{\prime} = - R_{c} \cos \phi^{\prime} + \sqrt {R^{2} - R_{c}^{2} \sin^{2} \phi^{\prime}} , \hfill \\ \end{gathered}$$

where *h*_LC_ is the thickness of the LC phase in the droplet (see Fig. 9c) and the director field described by Eqs. (2) and (3) is depicted in Fig. 9b, c, e, and f. Equation (2) has a singular axis at $r^{\prime} = 0$, where the director is along the z-axis, and the coordinate origin is set to the point where the singular axis crosses the bottom plane of the droplet. Strictly speaking, the field does not include defects with a discontinuous director. However, the director varies drastically near the I-N interface in the singular axis; we can consider that the point defect exists substantially at (*r*, *z*) = (0, *h*_LC_). Under this consideration, the parameter *R*_*c*_ in Eqs. (2) and (3) indicates the distance of the defect from the central axis of the droplet, which is consistent with the definition of *R*_*c*_ described in the previous section (inset of Fig. 3a). Using the Jones matrix method, we numerically calculated the light transmission intensity profiles under crossed polarisers, as shown in Fig. 9g and i. In both *R*_*c*_ = 0 and *R*_*c*_ > 0, the profiles were well consistent with the POM images (Fig. 9g–j).

The relation between *h*_LC_ and *T*_0_ can be estimated as follows:

4 $$\frac{{h - h_{LC} }}{h}\sim \frac{{T_{0} - T_{{{\text{NI}}}} }}{\Delta T} + \frac{1}{2},$$

where *T*_NI_ is the I-N transition temperature. In Fig. 3a and b, *R*_*c*_ shows non-zero value when *T*_0_ ranges from 41 to 45 °C; in this range ${{h_{{{\text{LC}}}} } \mathord{\left/ {\vphantom {{h_{{{\text{LC}}}} } h}} \right. \kern-0pt} h}$ shows the values between 0.2 and 0.6. In this situation, the flow field in the N phase region is dominated by the outwards flow, as shown in Fig. 9d. Because of this field, a structure with a centred point defect is destabilised.

Using Eqs. (1) and (2), we deduced the time evolution of *R*_*c*_. To adopt the Onsager variational principle, we calculated the Rayleighian ℜ composed of the dissipation function *W* and the time derivative of the free energy *F* ($\Re = W + \dot{F}$). In this case, *W* and *F* can be approximated as follows:

5 $$\begin{gathered} W = \gamma_{1} V_{s} Rh_{{{\text{LC}}}} \left[ {A\frac{{R_{c} }}{R} + B\left( {\frac{{R_{c} }}{R}} \right)^{3} } \right]\left( {\frac{{\dot{R}_{c} }}{R}} \right) + \gamma_{1} R^{2} h_{{{\text{LC}}}} X\left( {\frac{{\dot{R}_{c} }}{R}} \right)^{2} , \hfill \\ F = K_{3} h_{{{\text{LC}}}} \left[ {C\left( {\frac{{R_{c} }}{R}} \right)^{2} + D\left( {\frac{{R_{c} }}{R}} \right)^{4} } \right], \hfill \\ \end{gathered}$$

where $\dot{R}_{c}$ indicates the time derivative of *R*_*c*_, and terms independent of *R*_*c*_ are neglected. The non-dimensional parameters *A* and *B* depend on ${{h_{{{\text{LC}}}} } \mathord{\left/ {\vphantom {{h_{{{\text{LC}}}} } h}} \right. \kern-0pt} h}$, ${R \mathord{\left/ {\vphantom {R h}} \right. \kern-0pt} h}$ and ${{\gamma_{2} } \mathord{\left/ {\vphantom {{\gamma_{2} } {\gamma_{1} }}} \right. \kern-0pt} {\gamma_{1} }}$, where *γ*_1_ and *γ*_2_ are the rotational viscosities. *X* is a positive constant and *C* and *D* depend on ${{K_{1} } \mathord{\left/ {\vphantom {{K_{1} } {K_{3} }}} \right. \kern-0pt} {K_{3} }}$ and ${{K_{2} } \mathord{\left/ {\vphantom {{K_{2} } {K_{3} }}} \right. \kern-0pt} {K_{3} }}$, where *K*_1_, *K*_2_ and *K*_3_ are elastic constants^23,38,39,53,54^ (for more details, see Supplementary Note 8).

Minimising ℜ with respect to $\dot{R}_{c}$ according to the variational principle, we obtained the time evolution of *R*_*c*_ as follows:

6 $$\begin{gathered} \frac{{R_{c} }}{R} = \frac{{R_{c0} }}{R}\exp \left( { - \frac{t}{\tau }} \right)\left[ {1 + \frac{{(\gamma_{1} V_{s} RB + 4K_{3} D)R_{c0}^{2} }}{{(\gamma_{1} V_{s} RA + 2K_{3} C)R^{2} }}\left( {1 - \exp \left( { - \frac{2t}{\tau }} \right)} \right)} \right]^{{ - \frac{1}{2}}} \hfill \\ \tau = \frac{{2\gamma_{1} R^{2} X}}{{\gamma_{1} V_{s} RA + 2K_{3} C}}, \hfill \\ \end{gathered}$$

where *R*_*c*0_ is the initial value of *R*_*c*_ (*R*_*c*0_ = *R*_*c*_(*t* = 0)). In Eq. (6), as time *t* approaches infinity, *R*_*c*_ converges to zero or a non-zero value, depending on the sign of *τ*:

7 $$\begin{array}{*{20}c} {\frac{{R_{c} }}{R} \to 0} & {{\text{for}}\,\,\tau > 0} \\ {\frac{{R_{c} }}{R} \to \sqrt { - \frac{{\gamma_{1} V_{s} RA + 2K_{3} C}}{{\gamma_{1} V_{s} RB + 4K_{3} D}}} } & {{\text{for}}\,\,\tau < 0} \\ \end{array} .$$

Thus, the steady-state solution of *R*_*c*_ can exhibit a threshold behaviour. In addition, in both cases of Eq. (7), the time to reach steady state is characterised by the absolute value of *τ* defined in Eq. (6).

Using Eqs. (4), (6), and (7) and assuming that *V*_*s*_ is a constant of 1.0 μm/s, which is comparable to the measurement results of the flow field, we calculated the *T*_0_ dependence of the steady-state solution of ${{R_{c} } \mathord{\left/ {\vphantom {{R_{c} } R}} \right. \kern-0pt} R}$ and the absolute value of *τ*, as shown in Fig. 3c. ${{R_{c} } \mathord{\left/ {\vphantom {{R_{c} } R}} \right. \kern-0pt} R}$ shows a threshold behaviour, which is consistent with the behaviour observed in Fig. 3a and b. *τ* is of the order of 100 s and diverges near the threshold value. In Fig. 3a and b, temperature hysteresis of the order of 1 K is observed in ${{R_{c} } \mathord{\left/ {\vphantom {{R_{c} } R}} \right. \kern-0pt} R}$. Considering that the cooling and heating rates are 0.2 K/min, we can estimate that the characteristic time for the hysteresis behaviour is ~ 300 s, which is comparable with *τ* of the characteristic time for the structural deformation. This indicates that the deformation progresses sufficiently slowly compared to the rate of change in the temperature; this slowness is considered to result in a temperature hysteresis.

**Figure 10:**
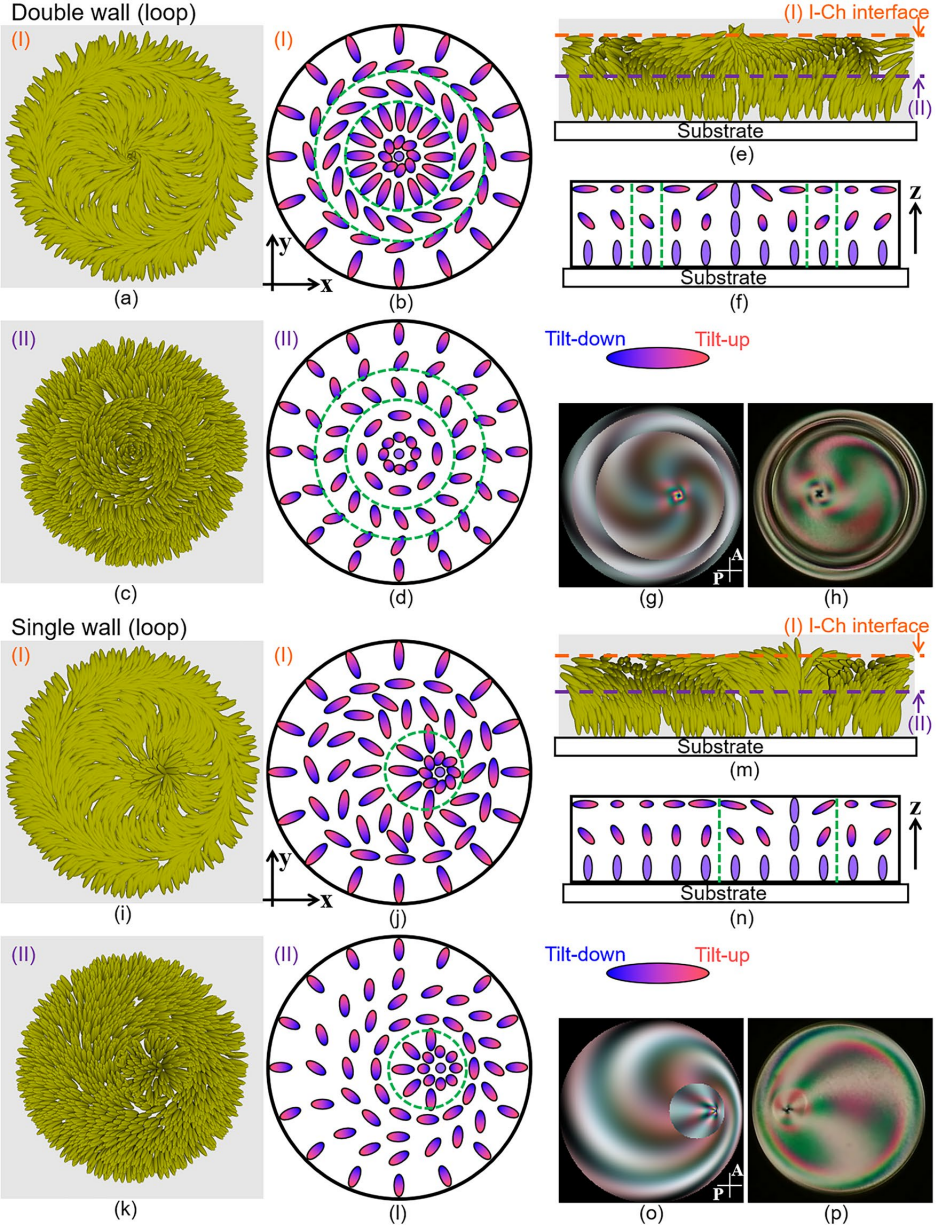
Analysis of director field in I + Ch phase. (**a**–**f**) represent the director field with double wall defects, and (**i**–**n**) represent the field with a single wall. (**e**), (**f**), (**m**) and (**n**) show the fields at the plane including the central axis of the droplet. (**a**), (**b**), (**i**) and (**j**) show the director fields at the plane indicated by the orange broken line (I) in (**e**) and (**m**), and (**c**), (**d**), (k) and (l) at the plane of the purple broken line (II). (**a**), (**c**), and (**e**) are depicted based on Eqs. (3), (S14), (S15a) and (S15b) in Supplementary Note 9, and (**i**), (**k**) and (**m**) are based on Eqs. (3), (S14), (S17a) and (S17b). In the schematics of (**b**), (**d**), (**f**), (**j**), (**l**) and (**n**), the colour gradation of the ellipsoids from red to blue represents the director tilt from up to down against the plane. The green broken lines/circles in the schematics indicate the position of the wall defects. Actually, the walls assumed in these director fields are considered to transform into the loop defects. (**g**) and (**o**) are the light transmission intensity profiles calculated by the Jones matrix method; (**g**) is based on equations (3), (S14), (S15a) and (S15b), and (**o**) is on equations (3), (S14), (S17a) and (S17b). P and A in (**g**) and (**o**) indicate polariser and analyser, respectively. (**h**) and (**o**) are POM images obtained just after the loop defects are formed; double and single loops exist in (**h**) and (**p**), respectively.

The director field in the I + Ch phase is also deduced. The present situation is similar to the Ch LC confined to the sandwich cells with strong homeotropic anchoring as reported in refs.^55–58^. According to these literatures, when the helical pitch length *P*_0_ and the cell thickness *d* is comparable with each other, the director fields can show the two states called “translationally invariant configuration” (TIC) and “cholesteric fingers” (CF). The difference of these states is in the distribution of the helical axis: in TIC the axis is uniformly parallel to the cell depth direction, while in CF the axis also distributes in the plane parallel to the substrates in addition to the depth direction. Similar to this situation, in our system *P*_0_ and *h*_LC_ are comparable with each other (see method section). Since the comma-like texture in relatively low *T*_0_ (Fig. 2g, h, m etc.) is similar to the pattern observed in CF, the helical axis parallel to the substrates is considered to be formed in this state. Here, we focus on the director field in relatively high *T*_0_, and discuss the field with a point defect as shown in Fig. 10. In this situation, a twisted cross-texture is often observed (e. g. Figure 2b and d), indicating that the director twists. We considered that this twist is attributed to the helical structure along the z-axis as similar to TIC, and that it gradually unwinds as *r*′ increases owing to homeotropic anchoring at the air interface (see Fig. 10a–f). Double- or single-loop defects were observed, in addition to helical structures (Fig. 4). For an intuitive understanding of the appearance of these defects, we considered the formation of cylindrical wall defects, which would actually deform into loops^23,59^. The wall resulted from the gap of the twist deformation along the z-axis; the twisting strength changed discontinuously at the wall (for more details, see Supplementary Note 9). Assuming that the director included double and single wall defects, we numerically calculated the light transmission intensity profiles under crossed polarisers, as shown in Fig. 10g and o, respectively. These profiles are well consistent with the POM images of Fig. 10h and p immediately after the double- and single-loop defects were formed, respectively. We consider that a field similar to that shown in Fig. 10 was formed in the I + Ch phase.

The stability of the defects is considered to depend on the parameter ${{R_{c} } \mathord{\left/ {\vphantom {{R_{c} } R}} \right. \kern-0pt} R}$. When ${{R_{c} } \mathord{\left/ {\vphantom {{R_{c} } R}} \right. \kern-0pt} R}$ is small, the double loop defects are formed (Fig. 4a–c); on the other hand, as ${{R_{c} } \mathord{\left/ {\vphantom {{R_{c} } R}} \right. \kern-0pt} R}$ increases the defects are destabilised and transformed into the single loop (Fig. 4d–f). In addition, ${{R_{c} } \mathord{\left/ {\vphantom {{R_{c} } R}} \right. \kern-0pt} R}$ shows the temperature hysteresis as shown in Fig. 3b. Owing to this hysteresis and the ${{R_{c} } \mathord{\left/ {\vphantom {{R_{c} } R}} \right. \kern-0pt} R}$-dependent stability property of the defects, the topologically different structures would be formed, depending on the temperature change process (Figs. 2 and 4).

In summary, we observed the formation of a director field in disk-shaped N and Ch droplets sandwiched by substrates under a temperature gradient. In both cases, during the coexistence phase, the field was largely deformed, showing hysteresis (Figs. 1, 2, 3). In the I + Ch phase, the deformation was accompanied by the rotation of the director field owing to the temperature gradient (Lehmann rotation). In addition, depending on the temperature change process, the formation and reconnection of loop defects were observed (Fig. 4). Owing to this topological variation, the final structures obtained in the Ch phase depend on the process.

Three-dimensional measurements of the flow field revealed the existence of the Marangoni convection. Convection was strongly induced in the entire droplet when the temperature was relatively high in the I + Ch phase, while it was suppressed and localised near the high-temperature region as the temperature decreased; in the Ch phase, the flow was hardly observed. We should consider that the Lehmann rotation in the I + Ch phase was mainly induced by the convection. This notion is consistent with the fact that the speed of the rotation in the I + Ch phase was higher by 1–2 orders of magnitude than the speed in the Ch phase, and that both direction of the convection and the rotation switched depending on the temperature (Figs. 5, 6, 7, 8). The appearance of the Marangoni convection also results in structural deformation with thermal hysteresis. By estimating the characteristic time of deformation and comparing it with the time of hysteresis, we found that both times exhibited the same order of magnitude. This indicates that the hysteresis behaviour was strongly associated with that the structural deformation was induced enough slowly, compared with the change rate of the temperature. This hysteresis property is considered to result in the topological variation of loop defects depending on the temperature change process.

As mentioned in the Introduction, the morphogenesis of living organisms proceeds in the presence of a potential gradient. To mimic this aspect, in this study, we focused on the structure formation of a Ch LC droplet under a temperature gradient and found that several nontrivial phenomena were induced. A remarkable fact is the simplicity of our system, in which only a typical Ch LC was used as the sample material. This suggests that a special gimmick would not be required to induce the phenomena: we expect that the Lehmann rotation and the topological change induced by flow are not specific phenomena induced in specific situations, but can be observed generally in the morphogenesis process of living organisms and soft matter systems, including the LC. Deoxyribonucleic acids and proteins, which strongly contributes to the morphogenesis, are chiral materials^1^. Since the morphogenesis is accompanied by the potential gradient, the rotation might be induced in these materials during the morphogenesis, as seen in the Lehmann rotation of Ch LC under the temperature gradient. In addition to the gradient, it has been reported that the flow is induced and plays significant roles in the morphogenesis^60–62^. Since it eventually needs the topological change as seen in segmentation and organogenesis, we expect that the flow might result also in the topological change of the living organisms, as observed in the LC droplet in this study.

## Methods

### Sample preparation

A rod-like molecule, 7CB (LCC Co., Ltd.), was used as the N LC. By adding the chiral dopant (S)-2-octyl 4-[4-(hexyloxy)benzoyloxy] benzoate (Tokyo Chemical Industry Co., Ltd.) to 7CB, we prepared Ch LC with a dopant concentration of 0.16 wt%. The phase sequences of the LC samples are N-42°C-I and Ch-42°C-I. The helical pitch length of the Ch LC sample was measured to be *P*_0_ ~ 50μm at 41 °C using the Cano-wedge method^23^. For fluorescence microscopy in the flow field measurement, the fluorescent dye poly[tris(2,5-bis(hexyloxy)-1,4-phenylenevinylene)-alt-(1,3-phenylenevinylene)] (Sigma-Aldrich Co., LLC) was added to the Ch LC sample.

To prepare sandwiched N LC and Ch LC droplets, we prepared cover glass substrates coated with CYTOP (Asahi Glass Co., Ltd.) for homeotropic alignment. By sandwiching the N LC and Ch LC samples with the substrates, we made the droplets whose side surfaces were exposed to air (see Figs. 1 and 2(v)). The distance between the glass substrates was maintained at 100 μm using polyimide film spacers, and droplets with diameters of 250–300 μm were used in this study (for more detail, see Supplementary Note 2).

### Temperature control and polarised microscopy

A temperature gradient was applied to the sample in a homemade furnace, as described in the literature^18^. The furnace consisted of a homemade hot stage and a commercial hot/cool stage (Tokai Hit Co., Ltd.). These stages respectively control the temperatures of the upper and lower substrates, touching the droplet (see Figs. 1 or 2(v)). In this study, the positive direction of the z axis was defined by the direction of the temperature gradient. The droplets were observed by polarised optical microscopy (POM) using a commercial upright microscope (ECLIPSE LV100, Nikon Co.).

### Surface tension measurement

Surface tension was measured using two methods. One is the pendant drop method, in which tension is determined by the size and shape of the pendant droplet^46,63^. The other one can be called ‘bubble method’, which is described in refs.^21,64,65^. We created a bubble using the LC samples and measured the pressure difference between the inside and outside of the bubble. Based on the Young–Laplace equation, we obtained the surface tension using the relationship between the pressure difference and radius of the bubble.

### Flow-field measurement by fluorescence photobleaching method

The flow field was measured using the previously described fluorescence photobleaching method^21,27,33^. The fluorescent LED illumination system D-LEDI (Nikon Co., Ltd.) was used as the light source for photobleaching. First, the sample was bleached into a striped pattern using strong light illumination through a photomask with a periodic array of slits. The flow distribution was obtained from the time evolution of the fluorescence images after photobleaching. Images were obtained using a confocal microscope system constructed with the MAICO MEMS confocal unit (Hamamatsu Photonics Co., Ltd.) and an inverted microscope Ti2-U (Nikon Co., Ltd.). The flow measurement was performed in each focal plane, as shown in Fig. 7, to realise a three-dimensional measurement of the flow field in the droplet. In this study, by defining a striped pattern aligned along the y-axis, we obtained the distribution of the flow velocity component in the x-direction, *v*_*x*_.

Setting the coordinate origin as the droplet centre, we considered that the observed 2D flow was a linear combination of radial and rotational flows. By defining the velocity components of the radial and azimuthal flows as *v*_*r*_ and *v*_*ϕ*_, respectively, we estimated them from the distribution of *v*_*x*_ obtained from the measurement. For simplicity, assuming that *v*_*r*_ and *v*_*ϕ*_ are independent of the azimuthal coordinates *ϕ*, we can describe *v*_*x*_ as

8 $$v_{x} (r,\phi ) = v_{r} (r)\cos \phi - v_{\phi } (r)\sin \phi .$$

From the *ϕ* dependence on *v*_*x*_, we estimated *v*_*r*_(*r*), as shown in Fig. 8 (for more details, see Supplementary Note 7).

### Analytical calculation and optical simulation

Analytical calculations were performed using the commercial software Mathematica (Wolfram Research, Inc.), and three-dimensional drawings of the director and flow fields based on the trial functions were created using the freely available POV-Ray software. An optical simulation based on trial functions of the director field was performed using the Jones matrix method^66^ (for more details, see ref.^18^). The ordinary refractive index of the sample was assumed to be 1.5^67^. For the birefringence index Δ*n*, we deduced Δ*n* of 7CB by measuring the transmission spectrum under crossed polarisers. The measurement was performed by a commercial spectroscope of BIM-6001A-06–Miniature Spectrometer (Brolight Technology Co., Ltd.), and the temperature was set to be 41 °C.

### Supplementary Information

Supplementary Information 1.

Supplementary Video 1.

Supplementary Video 2.

Supplementary Video 3.

Supplementary Video 4.

Supplementary Video 5.

Supplementary Video 6.

Supplementary Video 7.

Supplementary Video 8.

Supplementary Video 9.

## Data Availability

All data that support the findings in this study are available in the article and in Supplementary Information. Additional information is available from the corresponding author upon request.
