# Supplementary material for: Morphogenesis of a chiral liquid crystalline droplet with topological reconnection and Lehmann rotation
Source: Sci Rep. 2024 Mar 31;14:7597. doi: 10.1038/s41598-024-58054-2 (PMC11365937; doi:10.1038/s41598-024-58054-2)
Supplement: Supplementary file 1 — Supplementary Information 1. [file 41598_2024_58054_MOESM1_ESM.docx]

**Morphogenesis of a chiral liquid crystalline droplet with topological reconnection and Lehmann rotation**

Jun Yoshioka, Yuki Ito and Koji Fukao

**Supplementary Note:**

1. Legends for videos
2. Detail about experimental setup
3. Estimation of *T*_0_ and Δ*T*
4. Observation under equilibrium condition
5. Dependence of rotational speed on temperature gradient
6. Temperature dependence of surface tension
7. Estimation of radial flow velocity
8. Calculation result of Rayleighian in I+N phase
9. Assumption of director field in I+Ch phase

**1. Legends for videos**

**Video 1–3.** Structural formation of an N droplet. The detailed experimental condition is described in the legend of Fig. 1. Temperature difference between upper and lower glass substrates was Δ*T*=10K, and heating/cooling rate was 0.2K/min. The observation result in the 1st cooling process is shown in Supplementary Video 1, 1st heating and following 2nd cooling in Video 2, and 2nd heating in Video 3. The left and right videos in each are the observation results with crossed polariser and no polarizer, respectively. The playback speed is 60 times faster than real.

**Video 4–6.** Structural formation of a Ch droplet. The detailed experimental condition is described in the legend of Fig. 2. Temperature difference between upper and lower glass substrates was Δ*T*=10K, and heating/cooling rate was 0.2K/min. The observation result in the 1st cooling process is shown in Supplementary Video 4, 1st heating and following 2nd cooling in Video 5, and 2nd heating in Video 6. The left and right videos in each are the observation results with crossed polariser and no polarizer, respectively. The playback speed is 60 times faster than real.

**Video 7.** Formation of defect structure in 1st cooling process. The detailed experimental condition is described in the legend of Fig. 4. Temperature difference between upper and lower glass substrates was Δ*T*=10K, and heating/cooling rate was 0.2K/min. The video is shown with real-time speed. *T*_0_ changes from 43.8 °C to 43.7 °C.

**Video 8.** Reconnection of defect structure in the end of 1st heating processes. The detailed experimental condition is described in the legend of Fig. 4. Temperature difference between upper and lower glass substrates was Δ*T*=10K, and heating/cooling rate was 0.2K/min. The video is shown with 6 times faster than real. *T*_0_ changes from 44.1 °C to 44.5 °C.

**Video 9.** Director rotations under steady states. The detailed experimental condition is described in the legend of Fig. 5. Δ*T* is set to be 10 K, and *T*_0_ is 35.0 °C in (a) and (f), 37.5 °C in (b) and (g), 38.5 °C in (c) and (h), 42.0 °C in (d) and (i) and 43.0 °C in (e) and (j), respectively. The playback speed in (a) and (f) is 600 times faster than real, (b), (c), (g) and (h) are 60 times, and (d), (e), (i) and (j) are 6 times.

**2. Detail about experimental setup**

Schematics of the experimental setup is shown in Fig. S1. Sandwiching N or Ch LC samples by two cover glass substrates, we obtained oblate droplet. The substrates were coated by CYTOP (Asahi Glass Co., Ltd.) for homeotropic alignment. The side surface of the droplet is exposed to air, and the anchoring condition at the air interface is homeotropic. The distance between the substrates was maintained at 100μm, using polyimide film spacers, and the droplets with diameters of 250–300 μm were used in this study.

By heating and cooling the upper and the lower substrate respectively, we applied the temperature gradient to the droplet. The sapphire glasses were used because of its transparency and high heat conductivity, to realise microscope observations under temperature gradient. The observation was often performed under the coexistence state of the I and LC phases, as shown in Fig. S1. The I and LC phase regions locate in high- and low-temperature sides in the droplet, respectively.

**
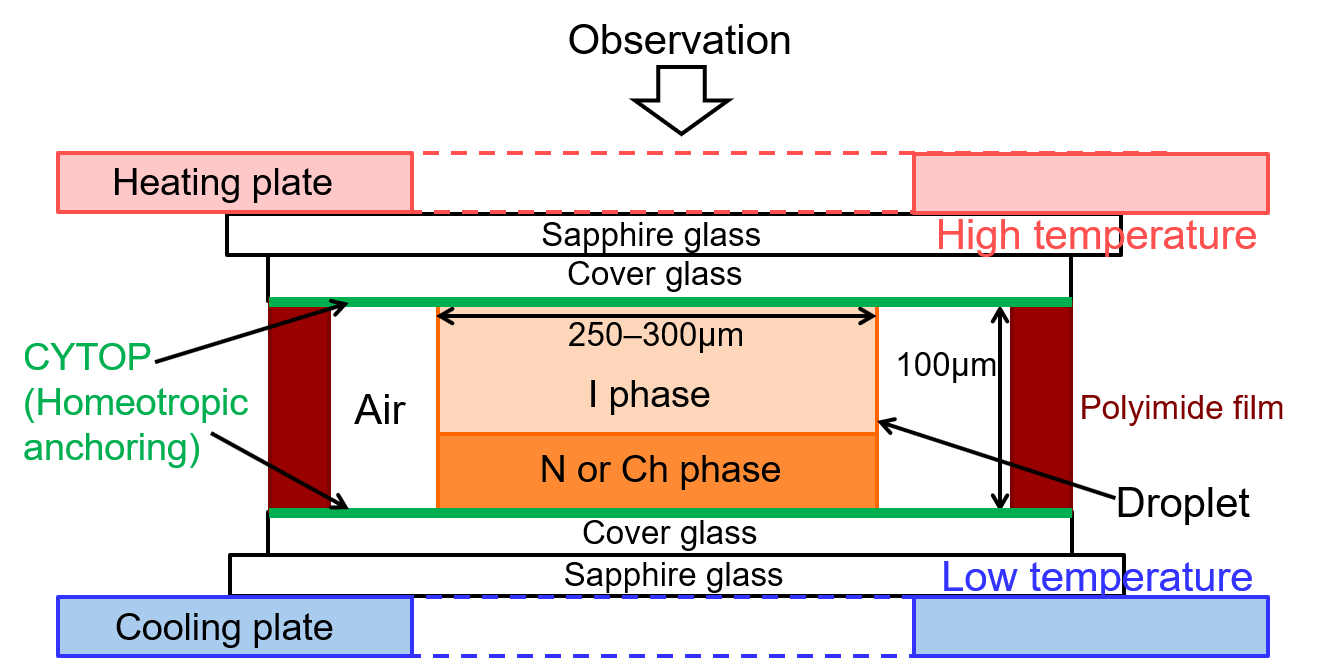
**

Fig. S1. Schematic image of experimental setup.

**3. Estimation of *T*_0_ and Δ*T***

We estimated *T*_0_ and Δ*T*, from the temperatures at the upper heating and lower cooling plates (see Fig. S1). These temperatures are defined as *T*_A_ and *T*_D_, respectively, which are the controllable parameters in the experiment. Defining that the temperature at the top of the droplet *T*_B_ and at the bottom as *T*_C_, we can describe *T*_0_ and Δ*T* as follows:

$T_{0}=\frac{T_{\text{B}}+T_{\text{C}}}{2}$, (S1a)

$\Delta T=T_{\text{B}}-T_{\text{C}}$. (S1b)

Assuming that the heat conductivity in the droplet is uniform and the effect of the latent heat is negligible, we simplify the present situation as shown in Fig. S2(a). The experimental system is composed of three regions of H, M and L, whose thicknesses are defined as *d*_1_, *d*_2_, and *d*_3_ respectively, and thermal conductivities are *λ*_1_, *λ*_2_, and *λ*_3_ respectively. These regions are connected linearly along z direction, and the temperature at the top of region H and the bottom of region L are set to be *T*_A_ and *T*_D_, respectively; the temperature at the border between region H and M is *T*_B_ and the temperature between M and L is *T*_C_. Applying the Fourier’s law [37] for the present situation, and considering that the heat flux density *q* is constant at the steady state in the case of one-dimensional heat conduction, we obtain,

$q=-\lambda_{1}\frac{T_{\text{A}}-T_{\text{B}}}{d_{1}}=-\lambda_{2}\frac{T_{\text{B}}-T_{\text{C}}}{d_{2}}=-\lambda_{3}\frac{T_{\text{C}}-T_{\text{D}}}{d_{3}}$. (S2)

Transforming Eq. (S2), we can describe *T*_B_ and *T*_C_ as,

$T_{\text{B}}=\frac{(1+\zeta_{2})T_{\text{A}}+\zeta_{1}T_{\text{D}}}{1+\zeta_{1}+\zeta_{2}}$, (S3a)

$T_{\text{C}}=\frac{\zeta_{2}T_{\text{A}}+(1+\zeta_{1})T_{\text{D}}}{1+\zeta_{1}+\zeta_{2}}$, (S3b)

where

$\zeta_{1}=\frac{\lambda_{2}d_{1}}{\lambda_{1}d_{2}}$, (S4a)

$\zeta_{2}=\frac{\lambda_{2}d_{3}}{\lambda_{3}d_{2}}$. (S4a)

Eqs. (S3a) and (S3b) indicate that *T*_B_ and *T*_C_ are determined if the parameters *ζ*_1_ and *ζ*_2_ are obtained.

To obtain *ζ*_1_ and *ζ*_2_, we use the special geometries shown in Figs. S2(b) and (c). We assume that the phase transition temperature *T*_t_ between the I and N/Ch phases at equilibrium agrees with the transition temperature under the temperature gradient. As described in the method section in the main text, *T*_t_ is 42 °C in this study. *T*_C_ agrees with *T*_t_ in Fig. S2(b), and *T*_B_ agrees with *T*_t_ in (c). Cooling the sample from the I phase under the existence of Δ*T*, the texture of LC start appearing under POM observation when the geometry shown by Fig. S2(b) is realised. The temperatures *T*_A_ and *T*_D_ at this situation is defined as *T*_A,I_ and *T*_D,I,_ respectively. We can also observe the textural change when the geometry shown by Fig. S2(c) is realised, and *T*_A_ and *T*_D_ under this situation is defined as *T*_A,Ch_ and *T*_D,Ch_, respectively. In the geometries of S2(b) and (c) the following relations are satisfied:

$T_{\text{C}}=T_{\text{t}}=\frac{\zeta_{2}T_{\text{A,I}}+(1+\zeta_{1})T_{\text{D,I}}}{1+\zeta_{1}+\zeta_{2}}$, (S5a)

$T_{\text{B}}=T_{\text{t}}=\frac{(1+\zeta_{2})T_{\text{A,Ch}}+\zeta_{1}T_{\text{D,Ch}}}{1+\zeta_{1}+\zeta_{2}}$ . (S5b)

For simplicity, we measured *T*_A,I_ *T*_D,I_, *T*_A,Ch_ and *T*_D,Ch_ under the constraint of,

$\Delta T_{\text{set}}\equiv T_{\text{A,I}}-T_{\text{D,I}}=T_{\text{A,Ch}}-T_{\text{D,Ch}}$. (S6)

Using (S5a), (S5b) and (S6), we obtain

$\zeta_{1}=\frac{T_{\text{A,Ch}}-T_{\text{t}}}{T_{\text{A,I}}-T_{\text{A,Ch}}}$ , (S7a)

$\zeta_{2}=\frac{T_{\text{t}}-T_{\text{D,I}}}{T_{\text{D,I}}-T_{\text{D,Ch}}}$ . (S7b)

Therefore, measuring *T*_A,I_, *T*_D,I_, *T*_A,Ch_ and *T*_D,Ch_, and using Eqs. (S1a), (S1b), (S3a), (S3b), (S7a) and (S7b), we can estimate *T*_0_ and Δ*T*. Based on this estimation, we set *T*_A_ and *T*_D_ in the experiment.

Fig. S2. (a) Simplified model about temperature distribution in experimental system. (b)(c) Geometries for the determination of the parameters *ζ*_1_ and *ζ*_2_. $T_{\text{C}}=T_{\text{t}}$ is satisfied in (b), and $T_{\text{B}}=T_{\text{t}}$ in (c).

L

M

H

*d*_1_

*d*_2_

*d*_3_

*T*_A_

*T*_B_

*T*_C_

*T*_D_

**z**

(a)

L

M

(I phase)

H

*T*_A,I_

*T*_t_

*T*_D,I_

L

M

(Ch phase)

H

*T*_A,Ch_

*T*_t_

*T*_D,Ch_

(b)

(c)

**4. Observation under equilibrium condition**

We realised type-A and -B states in the Ch phase using the protocol shown in Fig. 2, and after that we left them at room temperature, not applying the temperature gradient. As shown in Fig. S3(a) and (b), textural change was hardly observed in both Type-A and -B at least during 1 day, which was greatly larger than the characteristic time of the structure deformation *τ*, which was estimated in the order of 10^2^ seconds by equation (6) in the main text. In this estimation, *V_s_* is assumed to be zero because of the equilibrium condition. Since Type-A and -B states hold during the time much longer than *τ*, these structures are a stable or metastable structure at equilibrium.


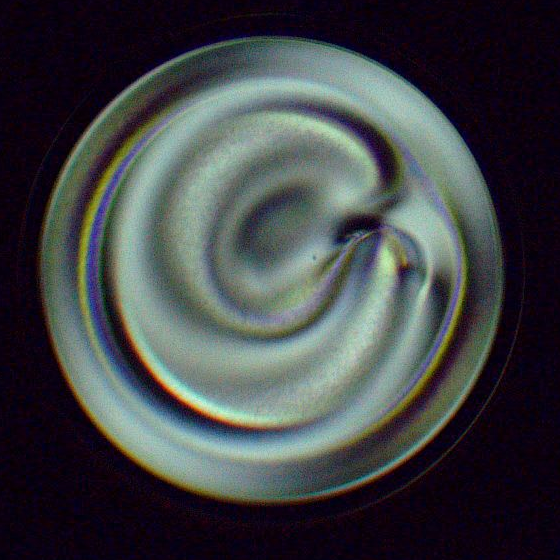

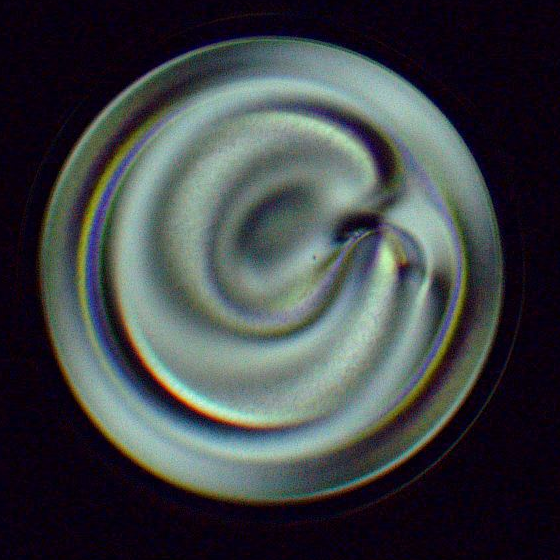


(a)

1 day


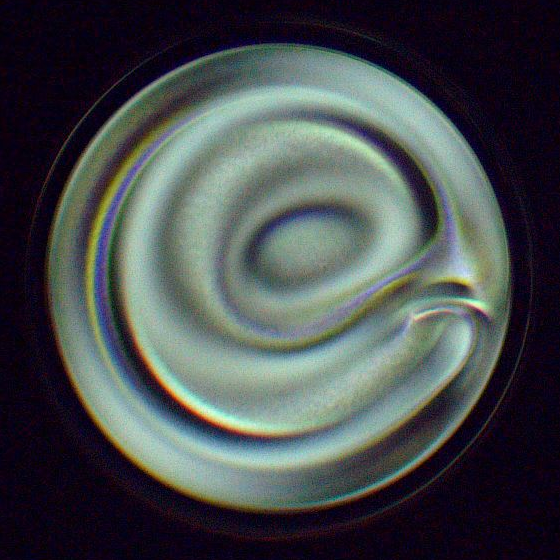

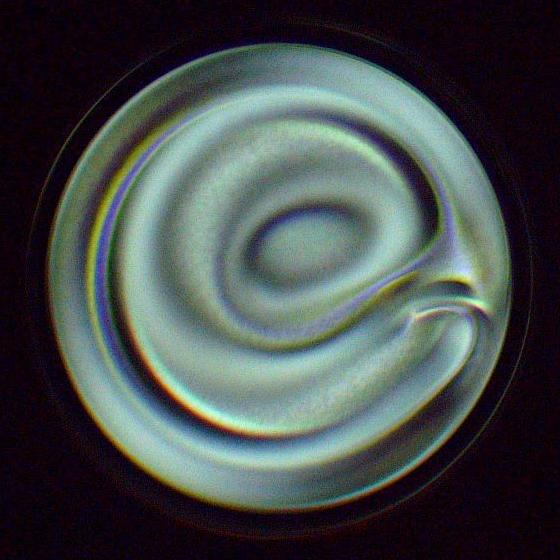


(b)

1 day

100μm

Fig. S3. Type-A and -B droplets under isothermal condition. (a) and (b) are the POM images of Type-A and B, respectively. The time interval in each image is 1 day. White bar in (a) indicates 100μm. *T*_0_ is set to the room temperature, and Δ*T* is zero.

**5. Dependence of rotational speed on temperature gradient**

In the Ch phase, Δ*T* dependence of the angular velocity of the director rotation was measured, as shown in Fig.S4. In both cases of Type-A and B the rotational speed was proportional to Δ*T*, while the speed of A was ~2 times higher than the speed of B.


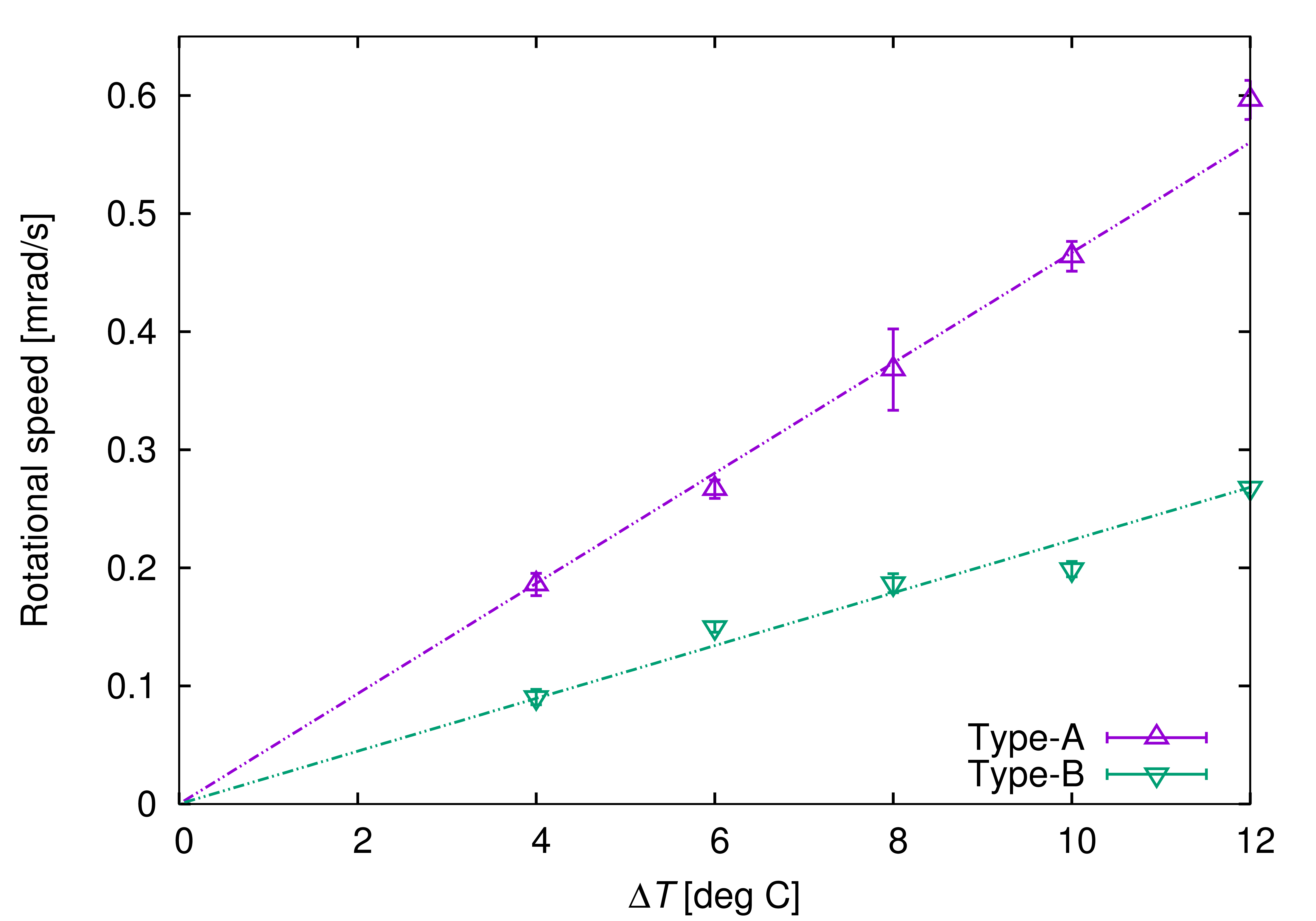


Fig. S4. Dependence of rotational speed on temperature difference. *T*_0_ was set to be 35.0 °C.

**6.** **Temperature dependence of surface tension**

The measurement results of the surface tension of Ch LC is shown in Fig. S5. When the temperature decreases, the tension decreases drastically at the transition point from the I to the Ch phase. In addition, the result obtained by the bubble method indicates that the surface tension gradually increases as the temperature decreases in the Ch phase. These properties of the temperature dependence of the surface tension is similar to those in 7CB [21, 43].


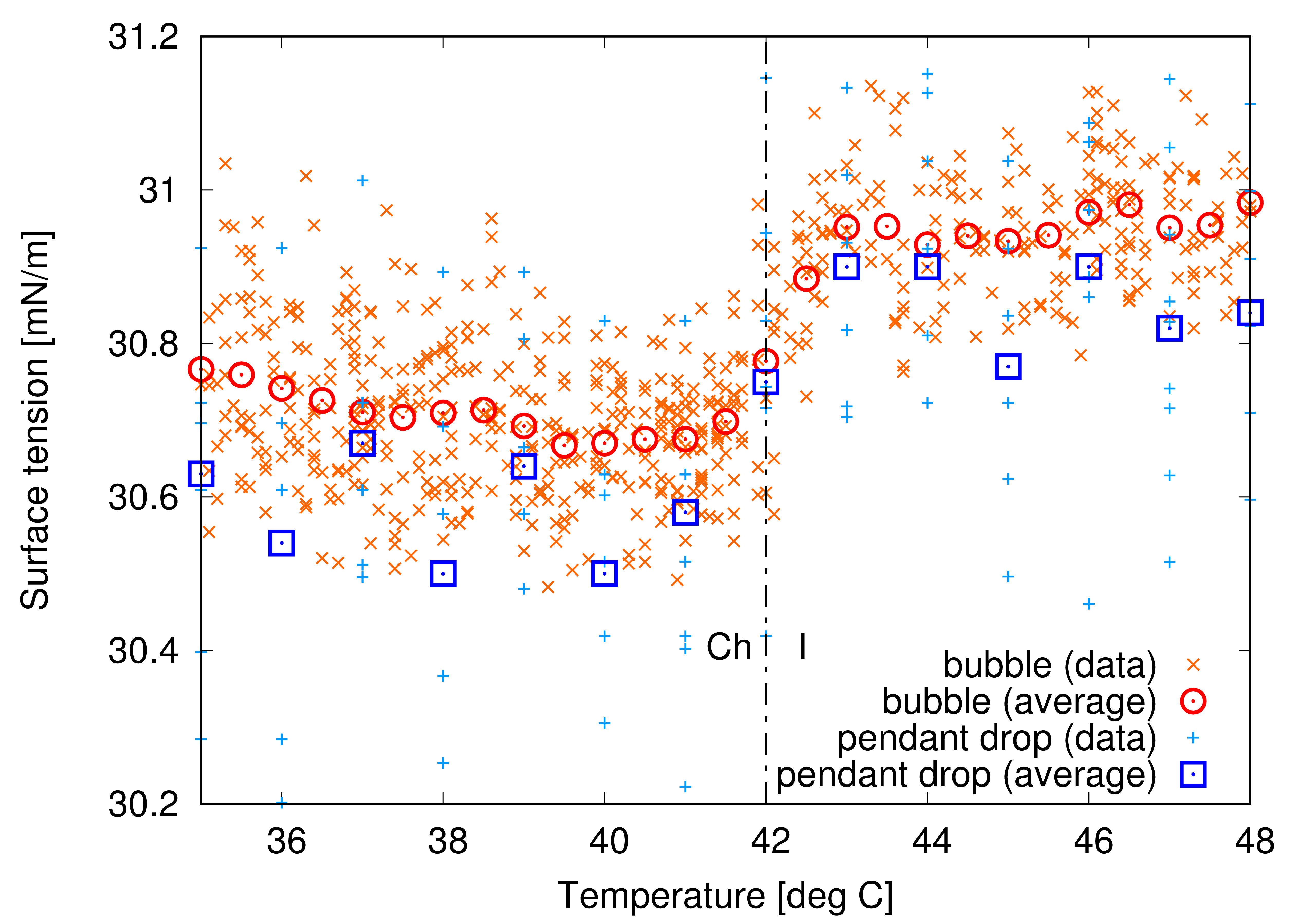


Fig. S5. Temperature dependence of surface tension in Ch LC. The sample is the mixture of 7CB and the chiral dopant, whose concentration was 0.16wt.% (see, Method section).

**7.** **Estimation of radial flow velocity**

Here we describe how the radial flow velocity *v_r_* was estimated from the distribution of the flow velocity component in the x direction, *v_x_*. As described in the method section, assuming that *v_r_* and *v_φ_* are independent of azimuthal coordinate *φ*, we can describe *v_x_* as

. (S8)

In this situation, *v_r_* and *v_φ_* should be regarded as constants under a fixed *r*. Thus, fitting the *φ* dependences of *v_x_* in respective *r* by the use of equation (S8), we obtained *v_r_* as shown in Fig. S6.


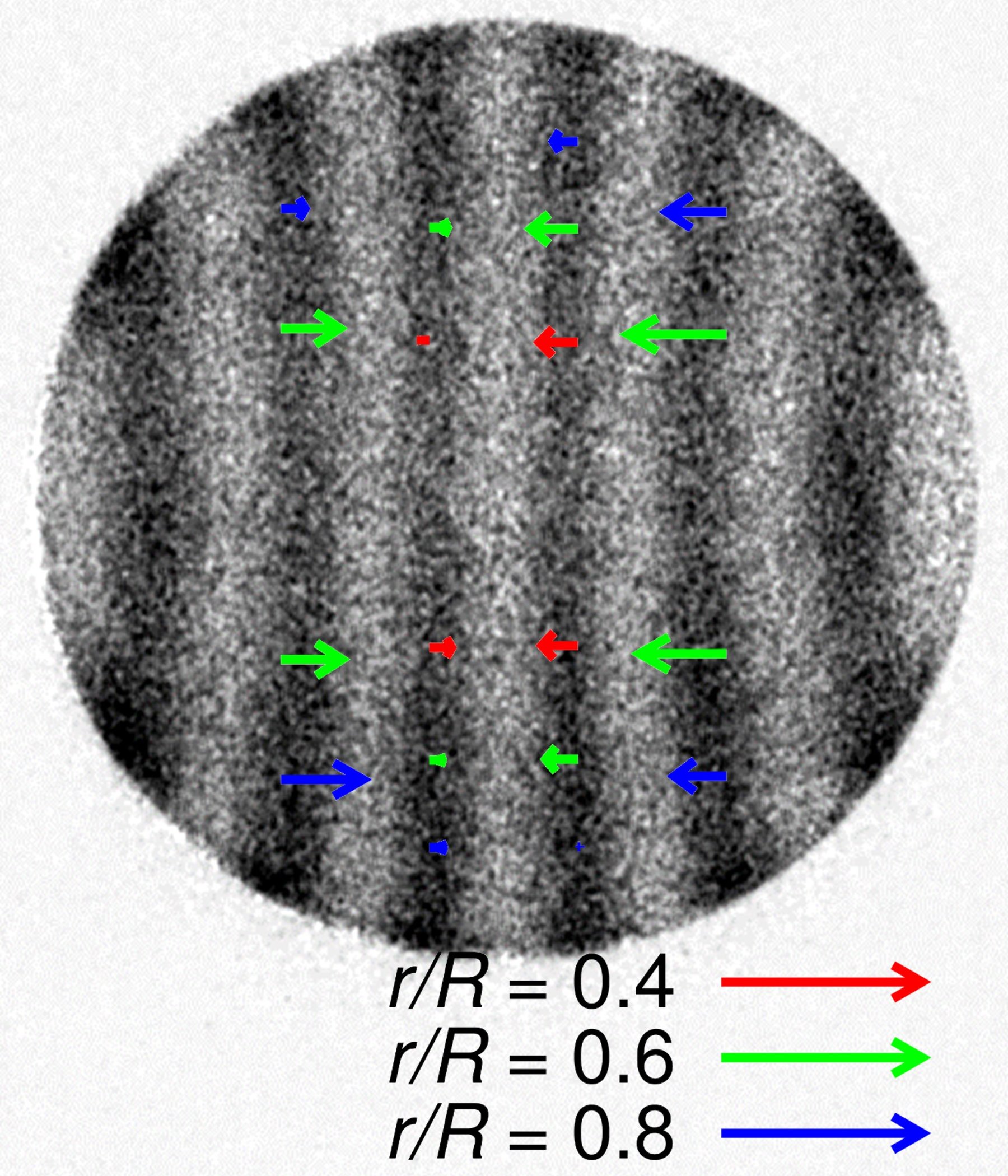

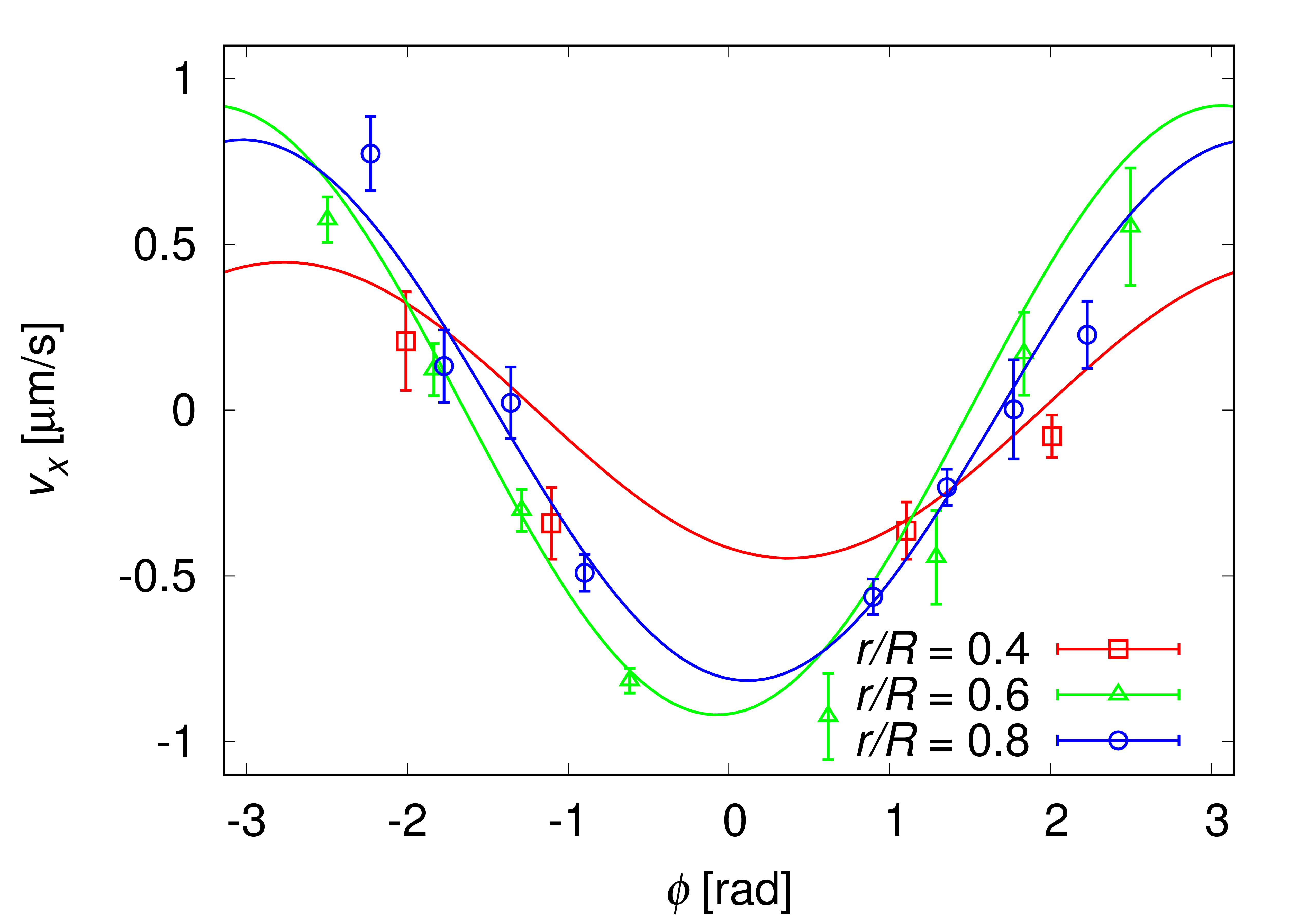


(a)

(b)

Fig. S6. Estimation of *v_r_* from the flow distribution obtained by the measurement. In (a), the obtained velocity components *v_x_* in the coordinates satisfying $r/R=$ 0.4, 0.6 and 0.8 are shown. *φ* dependences of the components were fitted by the use of equation S1 as shown in (b), so that we obtained *v_r_*.

**8. Calculation result of Rayleighian in I+N phase**

In N LC, the dissipation function per unit volume is described as,

$w=\frac{1}{2}\beta_{1}(e_{ij}n_{i}n_{j})^{2}+\frac{1}{2}\beta_{2}e_{ij}^{2}+\frac{1}{2}\beta_{3}\left(e_{ij}n_{j}\right)^{2}+\frac{1}{2}\gamma_{1}N_{i}^{2}+\gamma_{2}N_{i}e_{ij}n_{j}$, (S9)

$e_{ij}=\frac{1}{2}\left(\frac{\partial v_{i}}{\partial r_{j}}+\frac{\partial v_{j}}{\partial r_{i}}\right), N_{i}=\frac{\text{d}n_{i}}{\text{d}t}-\frac{1}{2}\left(\frac{\partial v_{i}}{\partial r_{j}}-\frac{\partial v_{j}}{\partial r_{i}}\right)n_{j}$, (S10)

where *i* and *j* denote the coordinates of *r*, *φ,* and *z*, and each component of the positional vector is denoted as *r_i_* (or *r_j_*). *β*_1_, *β*_2_, *β*_3_, *γ*_1_ and *γ*_2_ are the viscosity coefficients [38, 39, 50, 51].

The elastic free energy resulted from the director deformation is described as,

$f=\frac{1}{2}K_{1}(\text{div}\mathbf{n})^{2}+\frac{1}{2}K_{2}(\mathbf{n}\cdot\text{rot}\mathbf{n})^{2}+\frac{1}{2}K_{3}|\mathbf{n}\times\text{rot}\mathbf{n}|^{2}-\frac{1}{2}K_{24}\text{div(}\mathbf{n}\text{(div}\mathbf{n}\text{)}+\mathbf{n}\times\text{rot}\mathbf{n}\text{)}$, (S11)

where *K*_1_, *K*_2_, *K*_3_ and *K*_24_ are the elastic constants for splay, twist, bend, and saddle-splay deformations respectively [23].

Substituting equations (1)–(3) into (S9)–(S11), we integrated *w* and *f* in the cylinder with radius *R* and height *h*. Expanding them to fourth order of $R_{c}/R$, we obtained

. (S12)

Here, the parameters *A*, *B*, *X*, *C* and *D* are described as,

$\begin{matrix} A\sim(2.002-1.711\frac{\gamma_{2}}{\gamma_{1}})\frac{R^{2}}{h^{2}} \\ +\left((-40.17+8.852\frac{\gamma_{2}}{\gamma_{1}})\frac{R}{h}+(-7.599+6.218\frac{\gamma_{2}}{\gamma_{1}})\frac{R^{2}}{h^{2}}\right)\frac{h_{\text{LC}}}{h} \\ +\left((-3.573-2.859\frac{\gamma_{2}}{\gamma_{1}})+(90.62-20.09\frac{\gamma_{2}}{\gamma_{1}})\frac{R}{h}+(5.493-4.354\frac{\gamma_{2}}{\gamma_{1}})\frac{R^{2}}{h^{2}}\right)\frac{h_{\text{LC}}^{2}}{h^{2}} \\ +\left((5.617+4.398\frac{\gamma_{2}}{\gamma_{1}})+(-48.54+10.70\frac{\gamma_{2}}{\gamma_{1}})\frac{R}{h}\right)\frac{h_{\text{LC}}^{3}}{h^{3}} \\ +\left(-2.309-1.777\frac{\gamma_{2}}{\gamma_{1}}\right)\frac{h_{\text{LC}}^{4}}{h^{4}} \end{matrix}$ , (S13a)

$\begin{matrix} B\sim(-3.475+2.846\frac{\gamma_{2}}{\gamma_{1}})\frac{R^{2}}{h^{2}} \\ +\left((36.77-6.779\frac{\gamma_{2}}{\gamma_{1}})\frac{R}{h}+(12.66-9.713\frac{\gamma_{2}}{\gamma_{1}})\frac{R^{2}}{h^{2}}\right)\frac{h_{\text{LC}}}{h} \\ +\left((3.139+1.816\frac{\gamma_{2}}{\gamma_{1}})+(-83.38+15.21\frac{\gamma_{2}}{\gamma_{1}})\frac{R}{h}+(-8.901+6.489\frac{\gamma_{2}}{\gamma_{1}})\frac{R^{2}}{h^{2}}\right)\frac{h_{\text{LC}}^{2}}{h^{2}} \\ +\left((-4.732-2.511\frac{\gamma_{2}}{\gamma_{1}})+(44.78-8.023\frac{\gamma_{2}}{\gamma_{1}})\frac{R}{h}\right)\frac{h_{\text{LC}}^{3}}{h^{3}} \\ +\left(1.884+0.929\frac{\gamma_{2}}{\gamma_{1}}\right)\frac{h_{\text{LC}}^{4}}{h^{4}} \end{matrix}$, (S13b)

$X\sim1.759$, (S13c)

$C\sim0.1090\frac{K_{1}}{K_{3}}+0.9751\frac{K_{2}}{K_{3}}+1.256$, (S13d)

$D\sim0.08177\frac{K_{1}}{K_{3}}+0.2438\frac{K_{2}}{K_{3}}+0.3531$, (S13e)

where the numerical coefficients are shown with precision up to four digits. In the calculation, the approximations of $\sin\theta_{n}\sim\theta_{n}-\theta_{n}^{3}/6$ and $\cos\theta_{n}\sim1-\theta_{n}^{2}/2+\theta_{n}^{4}/24$ were used.

**9. Assumption of director field in I+Ch phase**

For the description of the director field in the I+Ch phase, we assumed the field as,

, (S14)

where *θ_n_* and *R*′ are defined by equation (3). The definition of *φ_n_* depends on whether the double or single wall-defects are formed. In the case of double wall, *φ_n_* is defined as,

, (S15a)

. (S15b)

*φ_n_* is related to the azimuthal angle of the director, and proportional to $z/h_{\text{LC}}$; this indicates the existence of the helical structure along z axis. On the cylindrical walls with $r'=\xi_{1}R', \xi_{2}R'$ the director is discontinuous. In the I-Ch interface of $z=h_{\text{LC}}$ as *r*′ goes to *ξ*_1_*R*′ or* ξ*_2_*R*′, equations (S8a) and (S8b) goes to the different values, which are respectively,

, (S16a)

, (S16b)

where the suffix *k* indicates 1 or 2. Therefore, on the cylinders of $r'=\xi_{1}R', \xi_{2}R'$, the director twist along z axis changes with *π* radians; this defect structure can be called twist-type *π*-walls [23, 56].

In the case of the single wall, *φ_n_* is defined as,

, (S17a)

. (S17b)

As well as the double wall, *φ_n_* is proportional to $z/h_{\text{LC}}$, indicating the existence of the helical structure along z axis. In the I-Ch interface, as $r'$ goes to$\zeta R'$, equations (S17a) and (S17b) also goes to the different values, which are respectively,

, (S18a)

. (S18b)

Therefore, the director field described by equations (17a) and (17b) has the *π*-wall on the cylinder of $r'=\zeta R'$.

Fig. 10 (a), (c), (e), (i), (k) and (m) are depicted based on equations (S14), (S15a), (S15b), (S17a) and (S17b). The existence of the *π*-walls described above can be observed in these figures.
